# Microsatellite Tandem Repeats Are Abundant in Human Promoters and Are Associated with Regulatory Elements

**DOI:** 10.1371/journal.pone.0054710

**Published:** 2013-02-06

**Authors:** Sterling Sawaya, Andrew Bagshaw, Emmanuel Buschiazzo, Pankaj Kumar, Shantanu Chowdhury, Michael A. Black, Neil Gemmell

**Affiliations:** 1 Centre for Reproduction and Genomics, Department of Anatomy, and Allan Wilson Centre for Molecular Ecology and Evolution, University of Otago, Dunedin, New Zealand; 2 Department of Pathology, University of Otago, Christchurch, New Zealand; 3 School of Natural Sciences, University of California Merced, Merced, California, United States of America; 4 G. N. Ramachandran Knowledge Centre for Genome Informatics, Delhi, India; 5 Proteomics and Structural Biology Unit, Institute of Genomics and Integrative Biology, Council of Scientific and Industrial Research, Delhi, India; 6 Department of Biochemistry, University of Otago, Dunedin, New Zealand; University of Pittsburgh, United States of America

## Abstract

Tandem repeats are genomic elements that are prone to changes in repeat number and are thus often polymorphic. These sequences are found at a high density at the start of human genes, in the gene’s promoter. Increasing empirical evidence suggests that length variation in these tandem repeats can affect gene regulation. One class of tandem repeats, known as microsatellites, rapidly alter in repeat number. Some of the genetic variation induced by microsatellites is known to result in phenotypic variation. Recently, our group developed a novel method for measuring the evolutionary conservation of microsatellites, and with it we discovered that human microsatellites near transcription start sites are often highly conserved. In this study, we examined the properties of microsatellites found in promoters. We found a high density of microsatellites at the start of genes. We showed that microsatellites are statistically associated with promoters using a wavelet analysis, which allowed us to test for associations on multiple scales and to control for other promoter related elements. Because promoter microsatellites tend to be G/C rich, we hypothesized that G/C rich regulatory elements may drive the association between microsatellites and promoters. Our results indicate that CpG islands, G-quadruplexes (G4) and untranslated regulatory regions have highly significant associations with microsatellites, but controlling for these elements in the analysis does not remove the association between microsatellites and promoters. Due to their intrinsic lability and their overlap with predicted functional elements, these results suggest that many promoter microsatellites have the potential to affect human phenotypes by generating mutations in regulatory elements, which may ultimately result in disease. We discuss the potential functions of human promoter microsatellites in this context.

## Introduction

Approximately 3% of the human genome is composed of microsatellites [1], tandem repeats composed of subunits between one and six nucleotides in length. During DNA replication, these sequences change in length at a rate that is many orders of magnitude higher than the average rate of point mutations [2]–[4]. Because microsatellites are often polymorphic, they have historically been used as markers for parentage and forensic analyses [5], [6]. Traditionally, microsatellites and other tandem repeats have been considered to be non-functional, neutral markers. However, there is increasing evidence that this is not always the case [7], [8]. For example, in the yeast genome, tandem repeats are frequently found in promoters and are directly responsible for divergence in transcription rates [9]. When tandem repeats within yeast promoters change in length, promoter structure and transcription factor binding can be altered [9], [10]. A similar process may occur in the human genome, where tandem repeats can also be found at a high density within promoters [9], defined here as 5 kilobases (kb) upstream and downstream of the transcription start site (TSS).

Recently, we identified human microsatellites that are conserved across vertebrate genomes [11], and later developed a phylogenetic method to measure this conservation [12]. We discovered that highly conserved mammalian microsatellites are over-represented in the promoter regions of various human genes, many of which regulate growth and development [12], [13]. Changes in the lengths of microsatellites within promoters can sometimes drastically alter phenotypes [7], [13]. For example, expansion of microsatellites in protein coding or 5′ untranslated regions (UTR) is well known to cause disease, including Huntington’s disease and fragile-X syndrome [7].

Microsatellites can also affect phenotypes when they are not transcribed [7], [13], [14]. By altering levels of gene expression, untranslated microsatellites proximal to a TSS can have significant effects on phenotypes. For example, a large body of work has linked variation in human phenotypes with regulatory microsatellites composed of the motif AC/GT [15]–[34]. Intriguingly, many of these studies focus on genes expressed in neuronal cells [15]–[21], such as PAX6 expression during eye development [20], [21] or NOS1 expression in the brain [15]–[17]. The promoters of neural development genes such as these contain a striking number of conserved microsatellites [12], [35].

Promoter microsatellites have the potential to form various DNA secondary structures, some of which are known to be involved in the regulation of gene expression [13], [36]. For example, microsatellites with the motif AC/GT can form Z-DNA, a left-handed spin double helix [37], and microsatellites composed of the motif AG/CT can form H-DNA, a DNA triplex [38]–[41]. Another DNA secondary structure of interest here is the G-quadruplex (G4, reviewed in [42]). G4 is predicted to form in sequences with the pattern (G
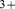
N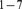)
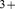
(G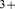) which due to its repetitive nature can be composed of microsatellites [43], such as (TGGG)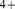 [44]. Formation of G4 induces single-strandedness in the complement C-rich strand, which can sometimes form an i-motif [42]. Predicted G4 sequences show a strong preference for promoter regions [45]–[48]. These structures can regulate transcription by modulating polymerase activity [49], [50] or by affecting RNA folding when present in 5′ UTR [51], [52].

To better understand how microsatellites are related to promoters and their various regulatory elements we used a wavelet analysis, adapted from ref. [53]. A wavelet decomposition transforms a signal into two components: detail coefficients and smooth coefficients. These coefficients have values at different scales, and these scales increase by a factor of two. The wavelet coefficients can be used to reconstruct the original data. The smoothed coefficients can be seen as similar to a weighted average of the signal, taken at multiple scales. If two signals are compared using smooth coefficients, the result is similar to that which would be found if their average densities were compared. If instead the details coefficients were compared, the result would be similar to comparing covariance between signals, because the detail coefficients measure the change in a signal [53]. Importantly, the wavelet coefficients at any single scale are independent (orthogonal) measures from the coefficients at the other scales [53]. This conveniently allows us to measure correlations between signals at multiple scales [53]–[55].

Our wavelet analysis included 32 non-continuous regions in the human genome, each 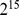 kb in length, for a total of 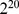 kb of DNA (approximately one billion bases). Wavelets are able to easily handle discontinuities in the data, such as those that are present between each of the 32 regions examined here [56]. We measured the densities of various elements across these regions, including those of canonical importance to promoters: GC content, protein coding regions and 5′ UTR. In addition, we examined two other factors known to be associated with promoters: predicted G4 regions [45]–[48] and CpG islands (CpG dinucleotide rich regions [57]). We focused on G/C rich promoter elements because promoter microsatellites tend to be G/C rich [58]. We examined the pair-wise relationship between all of these variables, and then using a linear model of wavelet coefficients, we examined how these different factors may interact to affect the association between microsatellites and promoters. The intention of the linear model of the wavelet coefficients was to determine if the significant association between microsatellites and promoters was caused by these other elements.

This is the first study to statistically test for an association between microsatellites and promoters. We discovered a highly significant, but complex relationship that depends heavily on microsatellite motif. In addition, we also found associations between microsatellites and the various promoter elements examined in the wavelet analysis. We discuss how microsatellite variation within these promoter elements may modulate gene expression, with a focus on DNA and RNA structure.

## Results and Discussion

### Microsatellite Motifs in Promoters

The most common microsatellite motifs in the human genome are A/T rich and more than a third of microsatellites in our data set (36.4%) are composed of the motifs A/T or AC/GT (Table 1). These two motifs are also the most common motifs within 5 kb of the TSS (Table 2). The third most common motif within the promoter region is CCG/CGG, but importantly, this motif is very uncommon in the genome, representing less than 1% of the microsatellites in our data set. In fact, of the 3820 CCG/CGG microsatellites we examined, 74% were found within 5 kb of the TSS. A similar motif, CCCG/CGGG, displayed the same preference for promoters, with 62% found within 5 kb of the TSS (Table 2). Intriguingly, microsatellites with the motif CCG/CGG are often very highly conserved in mammals, while the other G/C rich motifs are usually not conserved [12].

**Table 1:** Frequencies of motifs for all simple microsatellites in the human genome.

Motifs	Counts (freqency)
A/T	104,373 (19.4%)
AC/GT	91,786 (17.0%)
AT/TA	37,219 (6.91%)
AAAT/ATTT	30,771 (5.71%)
AAT/ATT	26,782 (4.97%)
AG/CT	23,680 (4.39%)
AAAC/GTTT	21,156 (3.92%)
AAC/GTT	17,974 (3.33%)
AATG/CATT	15,045 (2.79%)
AAAG/CTTT	14,865 (2.75%)
AAAAC/GTTTT	12,610 (2.33%)
AAGG/CCTT	10,681 (1.98%)
AGG/CCT	10,438 (1.93%)
AGGG/CTTT	10,314 (1.91%)
AGC/GCT	6,169 (1.14%)
CCG/CGG	3,820 (0.70%)
CCCG/CGGG	1,098 (0.20%)

The most common motifs in the human genome are shown, along with their counts and frequencies relative to all other microsatellites. A few motifs commonly found in promoters are also shown. The total number of microsatellites examined here is 538,964.

**Table 2:** Most common motifs found within 5 kb of the TSS and their strand-specific motif results.

Motifs	Counts (on coding strand)	Binom. p-value	KS Test Distance (p-value)
A/T	6559 (2803/3756)	5.2E−32	0.135 (<1E−300)
AC/GT	5072 (2051/3021)	2.1E−42	0.118 (3.1E−15)
CCG/CGG	2833 (1151/1682)	1.7E−23	0.06 (7.2E−3)
AAAT/ATTT	1419 (610/809)	1.4E−7	0.166 (9.1E−9)
AG/CT	1405 (686/719)	0.39	0.07 (0.042)
AGGG/CCCT	1308 (662/646)	0.68	0.07 (0.06)
AAT/ATT	1245 (577/668)	0.011	0.06 (0.15)
AGC/GCT	990 (373/617)	8.36E−15	0.134 (4.7E−4)
AAAC/GTTT	983 (434/549)	2.7E−4	0.188(6.4E−8)
AAC/GTT	952 (460/492)	0.315	0.182 (2.7E−7)
AATG/CATT	876 (452/424)	0.36	0.09 (0.055)
AAAG/CTTT	751 (325/426)	2.6E−4	0.084 (0.146)
AAAAC/GTTTT	651 (304/347)	0.10	0.137 (4.5E−3)
CCCG/CGGG	687 (274/413)	1.28E−7	0.114 (0.027)
AAGG/CCTT	659 (299/350)	0.050	0.092 (0.128)

The most common motifs and their strand-specific counts are displayed. The binomial test (Binom.) p-value is the chance that these strand-specific frequencies deviate from an expected value of 50%. The Kolmogorov-Smirnov (KS) test values provide a measurement of the difference between the distribution of the two different strand-specific motifs, for each motif pair. The p-values shown are not corrected for multiple tests.

### Linear Modeling of Distance to TSS

**Figure 1:**
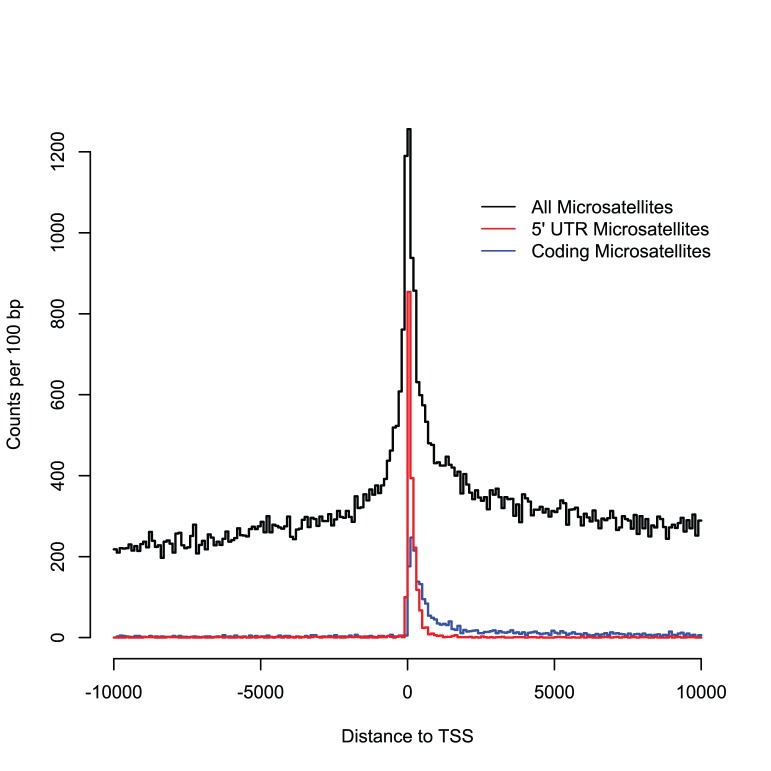
Distribution of microsatellites around promoters. The total number of microsatellites present in each 100 base-pair bin are provided for all microsatellites within 10 kb of the TSS. Also shown are the total number of only coding microsatellites (blue) or only 5′ UTR microsatellites (red).

There is a high density of microsatellites around the TSS of human genes (Figure 1). To determine which motifs show the strongest preference for the TSS, we used a linear model. For the response variable in this model we used distance to the nearest TSS, calculated for all microsatellites within 5 kb of the TSS, and we examined this variable in relation to motif for upstream and downstream regions separately. Table 3 displays the motifs with the strongest association to promoters for both upstream and downstream regions. G/C rich motifs have a strong association with promoters. Intriguingly, the most common motifs in the genome, mostly A/T rich, have a strong negative association with promoters. The intent of this model was to uncover the motifs with the strongest positive or negative relationship with distance to the TSS. We did not include overlap with functional elements, such as the 5′ UTR, or microsatellite length so that the results could be interpreted simply as the repeated motifs enriched or depleted around the TSS.

**Table 3:** Most significant motifs associated with distance to the TSS from the linear analysis.

Upstream: Motif	Sorted q-values	Reg.coef.
(Intercept): A/T	0.0E+00	−2.2E+03
CCG/CGG	2.7E−195	1.7E+03
CCCCG/CGGGG	2.1E−102	1.9E+03
CCCG/CGGG	1.2E−70	1.7E+03
AGG/CCT	2.7E−26	6.7E+02
CG/CG	5.6E−23	1.8E+03
C/G	3.2E−17	1.0E+03
CCCCCG/CGGGGG	1.3E−12	1.6E+03
AGGG/CCCT	6.7E−12	4.5E+02
CCGCG/CGCGG	7.5E−12	1.9E+03
CCCGG/CCGGG	1.5E−11	1.9E+03
AGCG/CGCT	3.4E−11	1.6E+03
AAAT/ATTT	1.9E−09	−3.7E+02
AT/AT	3.2E−09	−3.7E+02
AAT/ATT	7.9E−08	−3.4E+02
**Downstream: Motif**	**Sorted q-values**	**Reg.coef.**
(Intercept): A/T	0.0E+00	−2.5E+03
CCG/CGG	0.0E+00	2.0E+03
CCCG/CGGG	7.4E−165	1.9E+03
AGC/GCT	1.7E−122	1.3E+03
AGG/CCT	8.8E−71	8.8E+02
CCCCG/CGGGG	3.9E−52	1.8E+03
CCCGG/CCGGG	3.8E−39	2.1E+03
AGCG/CGCT	1.7E−35	2.1E+03
AGGG/CCCT	4.7E−31	6.5E+02
CG/CG	1.0E−21	1.7E+03
CCGG/CCGG	1.2E−21	1.7E+03
CCGCG/CGCGG	7.3E−19	2.0E+03
CCCCGG/CCGGGG	2.5E−17	2.0E+03
AGGGG/CCCCT	5.1E−12	8.8E+02
CCCCCG/CGGGGG	7.4E−12	1.6E+03

The top 10 most significant motifs associated with distance to TSS (in base-pairs), for the upstream and downstream regions, analyzed separately. These factors are sorted by their false discovery rate q-value (Sorted q-values). The size of the regression coefficient (Reg. coef.) indicates the strength of the association, with large positive coefficients belonging to motifs frequently found near the TSS. The full list of significant factors can be found in.

Tables S1 and S2.

### Potential Functions of Promoter Microsatellites

**Figure 2:**
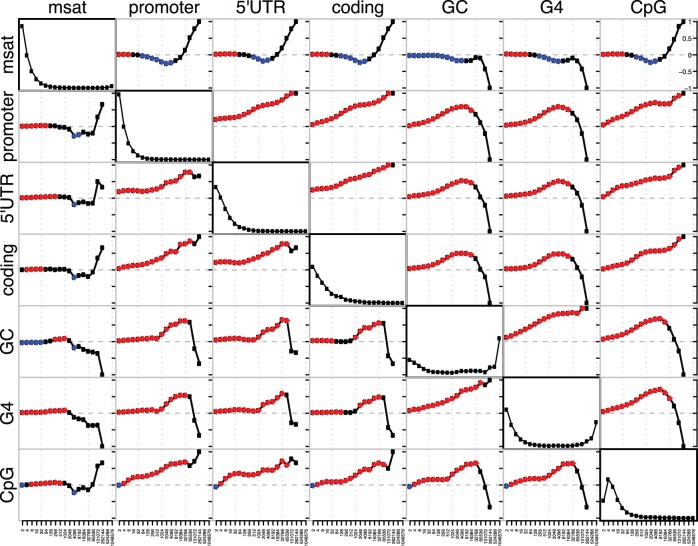
Kendall rank correlations between wavelet coefficients. The pairwise correlations between smooth coefficients are in the top right, and detail coefficients are the bottom left. The diagonal displays the normalized power spectrum for the wavelet coefficients, which can be interpreted as a measure of the variation of each signal at each scale. Note that the majority of factors examined here have most of their variation at the finest scales, while GC content and G4 elements contain a large amount of variation at the largest scales. Abbreviations for each element are “msat” for microsatellite, “G4” for predicted G4 regions, “CpG” for CpG islands, and “GC” for G/C content. Associations with a p-value above 0.001 are shown in red if positive, blue if negative. The smallest scale examined was 1 kb in size, and each successive scale increases by a factor of two.

**Figure 3:**
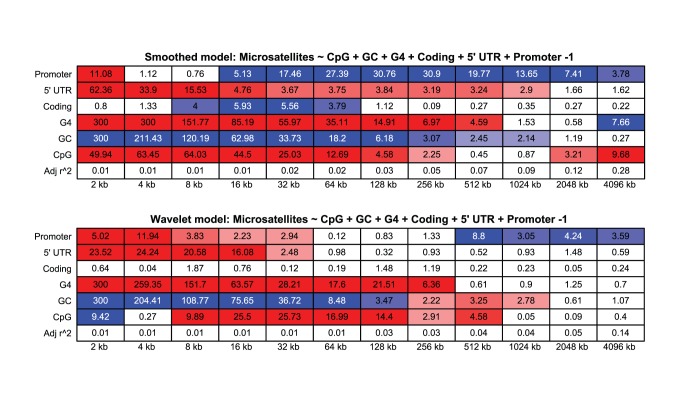
Linear model of wavelet results, displaying 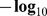 p-values. The top figure shows the results of the smooth coefficients, the bottom shows the results of the detail coefficients. Positive relationships are shown in red, negative in blue. The 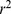 value is shown at the bottom of the figure. The largest scales were not included in this figure for simplicity.

As noted in a previous study of a subset of the human genome, there are many G/C rich microsatellites near the TSS of human genes [58]. Here we add that motifs with 100% G/C content are rarely found outside of promoter regions (Table 1) and are usually found very close to the TSS (Table 3). Many of these motifs have the potential to form various secondary structures [43], [59]. The G4 secondary structure is of particular interest to this study because there is increasing evidence that G4 elements play an important role in gene regulation [45], [46], [60]. These structures can be highly conserved in mammals [60], especially in promoter regions [45]–[47] and have been shown to modulate gene expression levels in microbes [61] and cancer cell lines [62]. Their prevalence in human gene promoters is particularly striking [45], [46] and our results support this observation (Figures 2, 3).

Many of the motifs found near the TSS have structural potential (Table 3). For example, the CCG/CGG motif can form secondary structures that are similar but not identical to canonical G4 structures [63], and changes in the length of these microsatellites have the potential to modulate gene expression [64] and cause disease when expanded [65]. A similar motif, CCCG/CGGG, is predicted to form G4 if repeated at least four times, and is similar to the GC-box, a transcription initiation site associated with the transcription factor SP1 [66]. Another motif that is predicted to form G4 DNA is AGGG/CCCT. This motif is common within promoters but is also relatively common elsewhere in the genome. Of the 10,314 AGGG/CCCT microsatellites, 1,308 of them are found within 5 kb of the TSS (Table 2).

G/C rich motifs that contain CpG dinucleotides are potential sites of epigenetic modification. Each of the 100% G/C microsatellites, except for the rare mononucleotide motif C/G, contain CpG dinucleotides [57]. Changes in repeat number for these CpG containing microsatellites would alter the number of potential methylation sites. However, changes in microsatellite length may also affect structural potential, which is important because G4 formation appears to restrict methylation at CpG dinucleotides [67]. So, although longer CpG containing microsatellites may contain more potential methylation sites, this may not directly translate into an increase in methylation because longer microsatellites may also have increased structural potential, and these structures may in turn interfere with methylation [68].

### Motifs on the Coding Strand

Transcription is most often uni-directional, with only one strand transcribed into RNA, leading to potential differences in sequence composition between the coding and non-coding strand. Therefore, we wondered if the microsatellite motifs on the coding strand might have different distributions around promoters than their counterparts on the opposite strand. Strand asymmetry exists between all non-palindromic motifs, and these motifs can be broken into pairs of strand-specific motifs. To examine how these strand-specific motifs are related to promoters, we obtained the microsatellite motifs on the coding (non-template) strand for the 37,249 microsatellites found within 5 kb of the TSS (Table 2).

**Figure 4:**
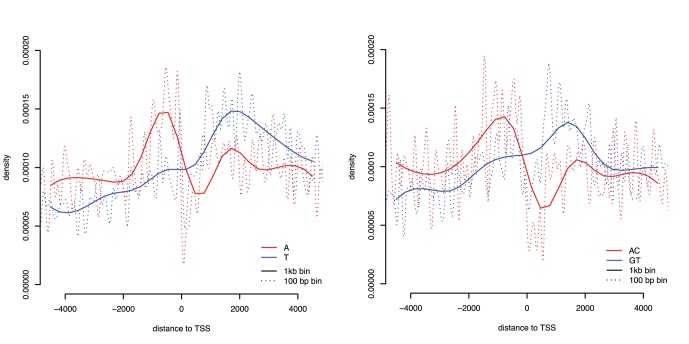
Strand-specific densities for the motifs A/T and AC/GT around promoters. These figures show the cubic spline of the densities of each strand-specific motif for bins of size 1kb (solid) and 100 base-pair (dashed) for the entire 5 kb promoter region.

The distributions for the most common strand-specific motif pairs, A/T and AC/GT are shown in Figure 4. These graphs show the smoothed density estimates for both 1 kb and 100 base pair bins. The strand-specific motifs A and AC display a preference for the upstream region and a depletion from the downstream region. Intriguingly, their counterparts, T and GT, display the complete opposite pattern, with their highest densities in the downstream regions. All of these motifs show depletion around the TSS, but this depletion is only clear when fine scale densities (100 base-pair bins) are examined.

Some of these strand-specific motifs have a preference for the coding strand (Table 2). For example, the motifs with 100% G/C (CCG/CGG and CCCG/CGGG) have a preference for the G-rich motif to be on the coding strand (59% and 60%, respectively). The binomial test p-values for these observations are 1.7E−23 and 1.28E−7, for CCG/CGG and CCCG/CGGG respectively. The other G-rich motifs common in promoters, AGGG/CCCT and AAGG/CCTT, do not show any preference for G-richness on the coding strand.

A strand-specific preference may be due to a selection for G-richness in RNA, and/or G-richness on the coding strand [69]. G-richness on the coding strand is also seen in predicted G4 forming regions around promoters [47]. Therefore, we were surprised that the predicted G4 motif AGGG/TCCC did not show any strong strand preference. The motif AG/CT, which is predicted to form H-DNA [38]–[41], also displayed no strand preference.

To examine whether the strand-specific distributions are different for each motif pair, we used the Kolmogorov-Smirnov test. The results of this non-parametric test indicate the distributions of many of these motif pairs are dissimilar to each other (Table 2). For example, the strand-specific motifs AC and GT have very different distributions around the TSS (Figure 4), and the Kolmogorov-Smirnov test results indicate this with a large distance value supported by a very low p-value. Notably, some motif pairs do not show any strand differences, such as the poly-purine/poly-pyrimidine motifs AG/CT, AAAG/CTTT and AAGG/CCTT.

Depletion of the motifs A and AC on the coding strand indicates that they may interfere with transcription (or translation when present in 5′ UTR). Perhaps this is unsurprising for the motif A, which is commonly known as a signal for the end of the transcript in the 3′ UTR, and may be selected against in the 5′ UTR. We are unaware of a similar explanation for the motif AC, which shows particularly strong depletion immediately downstream of the TSS. The Z-DNA structure that can form in AC/GT microsatellites is a left-handed double-helix with no known strand bias [37]. Changes in AC/GT length have been shown to modulate gene expression [70], as seen in the large number of studies associating AC/GT length variation with human phenotypes [15]–[34]. These strand-specific biases support the hypothesis that microsatellite motif can affect RNA structure [35], [71].

### Wavelet Analysis: Results on Multiple Scales

To statistically test for an association between microsatellites and promoters, we used a wavelet analysis on approximately one billion bases, a third of the entire genome. G/C rich motifs showed the strongest association with the TSS, so we wondered if the high density of microsatellites at the TSS (Figure 1) was caused by G/C rich regulatory elements. Therefore, in addition to promoters and microsatellites, we included various factors known to be associated with promoters: 5′ UTR, coding regions, predicted G4 regions [46]–[48], GC content, and CpG islands [57].

Figure 2 shows the pairwise Kendall rank correlations between each element at each scale for both the smooth and detail coefficients. Red indicates significant positive associations, and blue significant negative associations (p-value 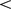 0.001). The power spectrum is shown on the diagonal, and represents the proportion of total variation explained by variation at each scale. Correlations between the smooth coefficients of these different elements (upper right portion of Figure 2) are functionally equivalent to correlations between average densities of these elements at various scales. The correlations between detail coefficients (bottom left portion of Figure 2) are more closely related to covariance between the signals [53].

The results of the pairwise comparisons indicate that promoters and microsatellites are significantly associated, but only on fine-scale measurements (Figure 2). At larger scales, microsatellites are negatively associated with promoters. We interpret these results as support for a local association between microsatellites and the TSS, but that microsatellites are, in general, found at higher densities in regions that do not contain promoters. This change in value between fine and coarse scales highlights the importance of examining multiple scales for associations between genomic elements, as processes acting at fine scales can be different from those acting at coarse scales [53]. Intriguingly, microsatellites display the same positive fine-scale and negative large-scale association with every factor examined except G/C content. The negative correlation between microsatellites and GC content highlights the fact that most microsatellites in the human genome are AT rich (Table 1).

Because G/C rich motifs are strongly associated with promoters and because many of these motifs have the potential to act as sites of DNA methylation or structural formation, we hypothesized that CpG islands or G4 forming regions could influence the apparent association between microsatellites and promoters seen in Figure 1. To investigate this we used linear modeling of the wavelet coefficients, again following methods of ref. [53] (Figure 3). This approach used the microsatellite wavelet coefficients as the response variable, and the wavelet coefficients for the other factors as covariates. The 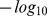 p-values are shown for each factor, at each scale. Again, significant positive associations are red, and negative associations are blue.

After controlling for these other factors, the relationship between promoters and microsatellites remained significant, but was again only positive at fine scales. Because fewer of the fine scales showed a significant positive association, the association between microsatellites and promoters at these scales can be partially attributed to the other factors examined. Intriguingly, the positive fine-scale associations between coding regions and microsatellites is absent when these other factors are considered.

The small 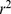 values here indicate that the total variance explained by this model is minimal. Therefore, there is a large amount of variation in microsatellite density that is not explained by these factors. Nevertheless, results of this linear model are highly informative and we stress that the intention of the model was not to determine which factors predict microsatellite density. Microsatellites are found throughout the genome, and hypothetically can arise and degrade by entirely neutral mutational processes [5], so we did not expect promoters and promoter-related factors to explain a large amount of variation in the microsatellite signal. We used this model to determine if the association between microsatellites and promoters was the result of a high density of GC rich elements around the TSS. Because the significant positive association between promoters and microsatellites remains when these other factors are included in the model, we can conclude that they are not entirely responsible for the high density of microsatellites found at the TSS (Figure 1).

### Relationship between Microsatellites and G4 Elements

The highly significant association between microsatellites and G4 supports the hypothesis that microsatellites sometimes play a role as structural elements [43]. In the pairwise comparison between G4 and microsatellite wavelet coefficients there is a highly significant association at fine scales (Figure 2), and this association increases when other factors are considered (Figure 3).

The motifs for microsatellites that overlap with G4 elements are shown in Table 4. Most of these motifs are similar to the canonical G4 definition but not all microsatellites with these G4-like motifs are considered G4 for two reasons. Some of these G4-like microsatellites are too short to have G4 potential (e.g., (AGGG)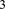). For longer microsatellites, we allow a few point mutations to disrupt the repeating pattern (i.e., they are imperfect repeats). If a point mutation disrupted the runs of adjacent guanines it would disrupt the G4 forming potential. Importantly, expansion of these G4-like microsatellites could result in novel G4 elements that would not present in the reference genome. For example, the G4-like microsatellite AGTG(AGGG)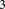 contains a point mutation that disrupts the perfect repeat and prevents G4 forming potential. This microsatellites could expand to form AGTG(AGGG)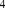, a microsatellite with G4 potential.

**Table 4:** Motifs of microsatellites that overlap with G4.

Motifs	Count	Avg. overlap (bp)	Avg. Overlap fraction
AGGG/CCCT	4610	16.9	0.85
ACCC/GGGT	1417	14.1	0.88
AGGGG/CCCCT	1114	25.9	0.85
C/G	961	18.0	0.98
ACCCC/GGGGT	585	18.6	0.92
CCCG/CGGG	583	14.0	0.86
CCCCG/CGGGG	485	19.6	0.88
AAGGG/CCCTT	427	27.5	0.79
AAGG/CCTT	352	8.4	0.21
AG/CT	306	9.1	0.23
AGCCC/GGGCT	293	19.7	0.87
AGGGC/GCCCT	264	19.4	0.86
AGG/CCT	236	10.7	0.36
ACCCCC/GGGGGT	234	22.1	0.92
AC/GT	176	4.8	0.17
CCG/CGG	157	7.2	0.31
AGCCCC/GGGGCT	154	21.4	0.78
CCCCCG/CGGGGG	116	21.5	0.88
CCCGG/CCGGG	106	19.5	0.88
AGAGGG/CCCTCT	93	24.0	0.70

Of the 13,838 microsatellites that overlap with a G4 element, the most common motifs are shown. For each microsatellite motif, the average base-pair overlap with G4 is shown (Avg. overlap (bp)). The average fraction of each microsatellite that overlaps with the G4 element is also shown (Avg. Overlap fraction). Note that motifs that are dissimilar to the canonical G4 definition, such as AC, usually share only a portion of the microsatellite in the G4 element.

As discussed above, some motifs have higher rates of expansion and contraction than others [72], [73], and therefore, some G4 and G4-like microsatellites will be more polymorphic than others. One motif in particular has a relatively high rate of expansion and contraction, the mononucleotide motif C/G [72]. Intriguingly, there are 1,402 C/G microsatellites in our data set and 961 (68.5%) overlap with a G4 element. G4 elements that overlap with these rare C/G microsatellites are expected to be highly variable.

Less variable G4 microsatellites may also be important because even small changes in repeat number for larger, G-rich motifs have the potential to alter secondary structure. Variation within G/C rich tandem repeats has been shown to affect gene expression and/or be associated with phenotypic differences in humans [64], [74]–[80]. For example, a CGGGGG/CCCCCG microsatellite in the ALOX5 gene has been repeatedly associated with cardiovascular disease [75]–[77]. Unfortunately, there is limited information about microsatellite variation available [81], even from the 1000 Genomes Project [82], so we are unsure exactly which G4 microsatellites contain variation that might affect structural potential. We expect recent advances in sequencing technology to help resolve this uncertainty [83].

To determine which pathways contain G4 elements that overlap with microsatellites, we used the Genomic Regions Enrichment of Annotations Tool (GREAT, [84]). This tool examines which genes contain a set of elements defined by the user (here G4 that overlap with microsatellites). To control for the fact that a limited sub-set of genes contain G4 elements within their promoters, we used the entire G4 set as a control group. Some of the results can be found in Table 5, and the rest are found in Table S4. Intriguingly, many of the genes that contain G4-microsatellites regulate cell signaling and/or development (Table 5).

**Table 5:** GO Results for genes with microsatellites that overlap with G4 elements.

Ontology	Category	Hyper FDR Q value	Hyper fold enrichment	Number of genes found
**GO Biological Process**	Signal release	3.39684e−7	2.1533	52
	Cartilage development	2.28192e−6	2.0690	41
	Negative regulation of B cell activation	5.84903e−5	4.4914	11
	Multicellular organismal homeostasis	1.20456e−4	2.1504	27
	Regulation of ion transmembrane transporter activity	1.87001e−4	2.1324	32
	Camera-type eye morphogenesis	5.21582e−4	2.0009	30
	Neurotransmitter secretion	5.19828e−4	2.1208	29
	Spinal cord anterior/posterior patterning	5.76268e−4	10.1377	1
	Tissue homeostasis	1.08506e−3	2.1131	21
	Regulation of long-term	1.25384e−3	3.0197	13
	neuronal synaptic plasticity			
	Hormone secretion	1.42278e−3	2.2221	22
	Hormone transport	1.76257e−3	2.1627	23
	Negative regulation of synaptic transmission, glutamatergic	3.77337e−3	6.1327	3
	Elevation of cytosolic calcium ion concentration involved in G-protein signaling coupled to IP3 second messenger	3.92996e−3	5.2566	5
	Peptide hormone secretion	4.22613e−3	2.2528	18
**PANTHER Pathway**	TGF-beta signaling pathway	4.57321e−4	2.0458	32
	General transcription regulation	6.12838e−3	3.1400	35
	Ras Pathway	6.46098e−3	2.0119	22
	Beta2 adrenergic receptor signaling pathway	2.56959e−2	2.0132	15
	Gamma-aminobutyric acid synthesis	2.90416e−2	4.7309	3
	Transcription regulation by bZIP transcription factor	3.47807e−2	2.0310	14

Gene ontology (GO) results for genes that contain microsatellites that overlap with G4 elements in their promoter. Hyper FDR Q-value is the false discovery rate q-value, Hyper fold enrichment is the enrichment of the test set on the overall (control) set for each category. 2,666 genes contain a G4 that overlaps with a microsatellite. For a control set we used genes that contain G4 elements in their promoters, for a total of 14,977 genes. The promoter region here was again 5 kb upstream and down of the TSS.

The relationship between microsatellites and G4 may have implications for quantitative genetics. Single nucleotide substitutions within predicted G4 regions can influence gene expression [85] and changes in microsatellite length within or around predicted G4 may be of equal or greater importance, as they would result in changes that are physically larger than single base changes. G4 microsatellites are potential sources of human phenotypic variation, and would make interesting candidates for association studies or molecular genetics experiments.

### Conclusions

The high density of microsatellites in promoters (Figure 1), together with their potential to function as structural elements [43], [59], suggests that some microsatellites can function as regulators of gene expression. Microsatellites are present in promoters more often than expected by chance. Promoter microsatellites are often G/C rich, and many promoter microsatellites are within or near 5′ UTR, CpG islands, and G4 structures. Variation within these promoter microsatellites has the potential to affect promoter function, which can ultimately lead to variation in phenotypes. This variation may be selectively beneficial [86], [87], and by targeting promoter microsatellites, especially those that are conserved [12], [71], we hope to uncover sources of human phenotypic variation.

## Materials and Methods

### Data

The microsatellite positions, their motifs, conservation and functional region (coding, 3 and 5-UTR, intronic, and intergenic) were taken directly from our previous work [11], [12], and we have previously released our data [12]. Our microsatellite definition is a tandem repeat composed of 1–6 base-pair motifs that is at least 12 nucleotides in length for motifs of length 1–4, and at least three uninterrupted repeats for motifs of length 5 and 6. As before, we only examined simple (non-adjacent) microsatellites on the autosomes that are found outside of transposable elements and duplicated regions. The positions for the CpG islands and the TSS (start of unique transcripts from the KnownCanonical table) were obtained from the UCSC genome browser [88]. To obtain the predicted G4 regions, we used the definition of G4 from ref. [60] and scanned the human genome (build 36/hg18) for unique (non-overlapping) G4 regions using the canonical G4 definition, (G
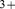
N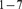)
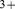
(G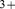) [45]. The positions for the 5′ UTR and coding regions of the human genome were obtained from Ensembl [89], [90]. The strand-specific motifs were obtained by taking the microsatellites found within 5 kb of the TSS, and analyzing the sequences on the coding strand. We detected microsatellites using SciRoKo [91], using the same parameters as we used in our previous work [11], [12].

### Linear Regression Analysis

Linear modeling was performed using the R statistical software package [92]. The response variable was the distance to the TSS, for microsatellites within 5 kb of the promoter, as defined by the start of the transcript in the KnownCanonical table from UCSC [88]. The covariate in this model was microsatellite motif (284 types). We corrected for multiple hypothesis testing by controlling the false discovery rate using the R package “fdrtool” and computed the false discovery rate q-value for each regression coefficient [93].

### Strand-specific Comparisons

To compare the distributions and counts of each strand-specific motif pair, we used a two tailed binomial test and a Kolmogorov-Smirnov test. Both of these tests were performed in R using default functions [92]. We did not correct for multiple tests here so that researchers interested in specific motifs can extract results independent of the other tests done.

### Wavelet Analysis

The methods and R code used for the wavelet analyses were adapted from ref. [53]. The value for each factor examined in the wavelet analysis was measured in 1 kb windows for each of the 32, 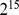 kb regions. For promoters, this regional measurement was a count of the number of promoters. For the other factors, this measurement was the total coverage in each of the 1 kb windows, as determined using the Galaxy [94]–[96] overlap tool. By examining coverage in each region, the length of each element is implicitly included in the model.

The regions we used cover 13 chromosomes, and the positions and brief description of each region can be found in Table S3. These regions were chosen because they are well annotated, and because they were used in a previous wavelet analysis on microsatellites [97]. The wavelet coefficients were generated for the entire set of regions, or 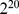 kb, and were scaled to preserve variance across scales.

To generate the wavelet coefficients, we used the Daubechies 4-tap wavelet transform, a slight variation from ref. [53], in which the Haar wavelet transform (Daubechies 2-tap) was used. Although we found similar results with other values for the Daubechies wavelet bases (results not shown), we chose the 4-tap basis because the results were more consistent between adjacent scales than the 2-tap bases, and it requires less computational time than the higher valued Daubechies transforms.

### Gene Ontology Analysis

GREAT 2.0.2 [84] was used for the gene ontology analysis. This web tool allows the user to input a set of genomic regions of interest (here G4 that overlap with microsatellites), and a control set on which to compare these regions (here all G4 regions). GREAT then tests the gene ontology categories which contain the regions of interest against the background set. It also corrects for false discovery rates. We used 5 kb upstream and downstream of the TSS as our promoter region.

## Supporting Information

Table S1 **Motifs significantly associated with upstream distance to transcription start site.**
(PDF)

Table S2 **Motifs significantly associated with downstream distance to transcription start site.**
(PDF)

Table S3 **Genome positions for the regions used in the wavelet analysis.**
(PDF)

Table S4 **Full table of GREAT analysis results.**
(PDF)
