# Supplementary material for: Microsatellite Tandem Repeats Are Abundant in Human Promoters and Are Associated with Regulatory Elements
Source: PLoS One. 2013 Feb 6;8(2):e54710. doi: 10.1371/journal.pone.0054710 (PMC3566118; doi:10.1371/journal.pone.0054710)
Supplement: Table S1 — Motifs significantly associated with upstream distance to transcription start site. (PDF) [file pone.0054710.s001.pdf]

Motif	q-values	Reg. coef.
(Intercept): A	0.0E+00	-2.2E+03
CCG	2.7E-195	1.7E+03
CCCCG	2.1E-102	1.9E+03
CCCG	1.2E-70	1.7E+03
AGG	2.7E-26	6.7E+02
CG	5.6E-23	1.8E+03
C	3.2E-17	1.0E+03
CCCCCG	1.3E-12	1.6E+03
AGGG	6.7E-12	4.5E+02
CCGCG	7.5E-12	1.9E+03
CCCGG	1.5E-11	1.9E+03
AGCG	3.4E-11	1.6E+03
AAAT	1.9E-09	-3.7E+02
AT	3.2E-09	-3.7E+02
AAT	7.9E-08	-3.4E+02
ACGC	1.9E-07	1.4E+03
CCGG	2.3E-06	1.4E+03
AGGGG	1.1E-05	7.1E+02
CCCCGG	1.8E-05	1.5E+03
ATAG	1.9E-05	-6.8E+02
ATC	4.4E-04	-4.8E+02
AAAC	4.9E-04	-2.7E+02
AGGCG	4.9E-04	1.6E+03
AGGCGG	5.9E-04	1.8E+03
ACTC	1.2E-03	6.1E+02
ACCCC	2.3E-03	6.7E+02
AGCC	3.4E-03	5.0E+02
AAC	4.0E-03	-2.2E+02
CCCGCG	5.4E-03	1.8E+03
AATT	5.8E-03	-4.2E+02
AGCGG	6.1E-03	2.0E+03
AGGGGG	8.1E-03	1.4E+03

Table S1. Motifs significantly associated with upstream distance to transcription start site. Only factors with significant q-values are shown. Regression coefficients (reg. coeff.) are also shown. These motifs do not represent strand-specific sequences. For example, the motif CCG is equivalent to CCG/CGG, and A is equivalent to A/T.
