# Supplementary material for: Microsatellite Tandem Repeats Are Abundant in Human Promoters and Are Associated with Regulatory Elements
Source: PLoS One. 2013 Feb 6;8(2):e54710. doi: 10.1371/journal.pone.0054710 (PMC3566118; doi:10.1371/journal.pone.0054710)
Supplement: Table S2 — Motifs significantly associated with downstream distance to transcription start site. (PDF) [file pone.0054710.s002.pdf]

Motif	q-values	Reg. coef.
(Intercept): A	0.0E+00	-2.5E+03
CCG	0.0E+00	2.0E+03
CCCG	7.4E-165	1.9E+03
AGC	1.7E-122	1.3E+03
AGG	8.8E-71	8.8E+02
CCCCG	3.9E-52	1.8E+03
CCCGG	3.8E-39	2.1E+03
AGCG	1.7E-35	2.1E+03
AGGG	4.7E-31	6.5E+02
CG	1.0E-21	1.7E+03
CCGG	1.2E-21	1.7E+03
CCGCG	7.3E-19	2.0E+03
CCCCGG	2.5E-17	2.0E+03
AGGGG	5.1E-12	8.8E+02
CCCCCG	7.4E-12	1.6E+03
ACG	1.4E-10	1.8E+03
ACGG	1.5E-09	1.9E+03
AT	4.5E-09	-3.5E+02
C	5.4E-09	6.3E+02
AGCCGG	2.2E-08	2.2E+03
AGCGGC	5.3E-08	1.8E+03
AGCCG	9.2E-08	2.2E+03
ACGC	1.3E-07	1.3E+03
AGCC	2.2E-07	6.3E+02
AGCCC	8.5E-07	8.2E+02
AGCCCC	9.7E-07	1.3E+03
AG	1.7E-06	2.7E+02
CCCGCG	2.7E-06	2.0E+03
AGCGG	4.2E-06	2.3E+03
AGGC	7.7E-06	4.2E+02
ACC	1.7E-05	3.5E+02
AAT	2.5E-05	-2.7E+02
AGCCGC	4.3E-05	2.2E+03
ACCCC	1.1E-04	6.0E+02
AGCCCG	1.3E-04	2.2E+03
ACCG	2.8E-04	1.8E+03
AGGCG	3.5E-04	1.9E+03
ACGGG	7.7E-04	1.6E+03
AGCTCC	7.7E-04	1.7E+03
ACCCCC	1.3E-03	7.2E+02
AAAT	1.4E-03	-1.9E+02
ACCC	1.5E-03	3.1E+02
AAATT	1.8E-03	-8.6E+02
AAAAT	1.8E-03	-4.0E+02
AGCGC	1.8E-03	2.2E+03
ACCGCC	2.3E-03	2.0E+03
AAGC	2.7E-03	5.8E+02
ATAC	3.5E-03	-3.7E+02
AGGCGC	4.3E-03	2.4E+03

Table S1. Motifs significantly associated with downstream distance to transcription start site. Regression coefficients (reg. coeff.) are also shown. These motifs do not represent strand-specific sequences. For example, the motif CCG is equivalent to CCG/CGG, and A is equivalent to A/T.
