# Supplementary material for: Microsatellite Tandem Repeats Are Abundant in Human Promoters and Are Associated with Regulatory Elements
Source: PLoS One. 2013 Feb 6;8(2):e54710. doi: 10.1371/journal.pone.0054710 (PMC3566118; doi:10.1371/journal.pone.0054710)
Supplement: Table S3 — Genome positions for the regions used in the wavelet analysis. (PDF) [file pone.0054710.s003.pdf]

Chromosome	Start	End	Description
1	29801000	62569000	mid short arm
1	62569000	95337000	mid short arm
1	147777000	180545000	mid long arm
2	21038000	53806000	mid short arm
2	53806000	86574000	short arm near centromere
2	111009000	143777000	mid long arm
2	149499000	182267000	mid long arm
2	182267000	215035000	mid long arm
3	47000	32815000	short arm near telomere
3	32815000	65583000	mid short arm
3	94988000	127756000	long arm near centromere
3	127756000	160524000	mid long arm
3	160524000	193292000	long arm near telomere
4	75672000	108440000	mid long arm
4	108440000	141208000	mid long arm
5	74000	32842000	short arm near telomere
5	49442000	82210000	long arm near centromere
5	97613000	130381000	mid long arm
6	62237000	95005000	long arm near centromere
6	95938000	128706000	mid long arm
7	478000	33246000	short arm near telomere
7	74604000	107372000	mid long arm
8	48310000	81078000	long arm near centromere
8	86852000	119620000	mid long arm
9	37000	32805000	short arm near telomere
9	91719000	124487000	mid long arm
10	81242000	114010000	mid long arm
11	1170000	33938000	short arm near telomere
11	95943000	128711000	long arm near telomere
12	36143000	68911000	long arm near centromere
12	75042000	107810000	mid long arm
13	17921000	50689000	long arm near centromere
13	50689000	83457000	mid long arm

Table S1. Position of the regions used in the wavelet analysis. These positions are for genome build hg18. A short description of each region is also included.
