# Supplementary material for: Microsatellite Tandem Repeats Are Abundant in Human Promoters and Are Associated with Regulatory Elements
Source: PLoS One. 2013 Feb 6;8(2):e54710. doi: 10.1371/journal.pone.0054710 (PMC3566118; doi:10.1371/journal.pone.0054710)
Supplement: Table S4 — Full table of GREAT analysis results. (PDF) [file pone.0054710.s004.pdf]

Table S4: Full Gene ontology results from GREAT analysis
From Sawaya et al. PLOS ONE

GREAT Table Output: Genomic Regions Enrichment of Annotations Tool, Bejerano Lab, Stanford University

Ontology	# Term Name	Hyper Rank	Hyper Raw P-Value	Hyper FDR Q-Val	Hyper Fold Enrichment	Hyper Foreground Region Hits	Hyper Total Regions	Hyper Region Set Coverage	Hyper Foreground Gene Hits	Total Genes Annotated
GO Molecular Function	cell adhesion molecule binding	17	7.02521e-6	1.40587e-3	2.5962	27	246	0.18%	21	50
	activating transcription factor binding	19	1.58066e-5	2.83022e-3	2.9568	20	160	0.13%	14	29
	extracellular matrix constituent, lubricant activity	21	6.55555e-5	1.06200e-2	4.9510	9	43	0.06%	2	5
	oxidoreductase activity, acting on paired donors, with incorporation or reduction of molecular oxygen, 2-oxoglutarate as one donor, and incorporation of one atom each of oxygen into both donors	22	7.04751e-5	1.08980e-2	2.8386	18	150	0.12%	14	46
	RNA polymerase II transcription factor binding transcription factor activity	24	8.45354e-5	1.19829e-2	2.4397	23	223	0.15%	17	57
	RNA polymerase II transcription cofactor activity	25	9.80460e-5	1.33421e-2	2.5344	21	196	0.14%	15	52
	cadherin binding	28	1.27618e-4	1.55056e-2	3.1843	14	104	0.09%	10	23
	phospholipase binding	30	1.96870e-4	2.23251e-2	3.9424	10	60	0.07%	6	13
GO Biological Process	signal release	97	3.76048e-9	3.39684e-7	2.1533	69	758	0.46%	52	131
	cartilage development	112	2.91686e-8	2.28192e-6	2.0690	67	766	0.45%	41	119
	negative regulation of B cell activation	151	1.00799e-6	5.84903e-5	4.4914	15	79	0.10%	11	25
	multicellular organismal homeostasis	166	2.28208e-6	1.20456e-4	2.1504	44	484	0.29%	27	103
	regulation of ion transmembrane transporter activity	171	3.64953e-6	1.87001e-4	2.1324	43	477	0.29%	32	77
	camera-type eye morphogenesis	190	1.13103e-5	5.21582e-4	2.0009	45	532	0.30%	30	79
	neurotransmitter secretion	191	1.13316e-5	5.19828e-4	2.1208	39	435	0.26%	29	69
	spinal cord anterior/posterior patterning	194	1.27592e-5	5.76268e-4	10.1377	6	14	0.04%	1	2
	tissue homeostasis	209	2.58820e-5	1.08506e-3	2.1131	36	403	0.24%	21	76
	regulation of long-term neuronal synaptic plasticity	216	3.09096e-5	1.25384e-3	3.0197	18	141	0.12%	13	25
	hormone secretion	217	3.52367e-5	1.42278e-3	2.2221	31	330	0.21%	22	56
	hormone transport	224	4.50599e-5	1.76257e-3	2.1627	32	350	0.21%	23	62
	negative regulation of synaptic transmission, glutamatergic	235	1.01203e-4	3.77337e-3	6.1327	7	27	0.05%	3	8
	elevation of cytosolic calcium ion concentration involved in G-protein signaling coupled to IP3 second messenger	237	1.06300e-4	3.92996e-3	5.2566	8	36	0.05%	5	12
	peptide hormone secretion	238	1.14793e-4	4.22613e-3	2.2528	26	273	0.17%	18	45
	cellular response to interleukin-6	242	1.30013e-4	4.70733e-3	5.9137	7	28	0.05%	5	8
	epithelial structure maintenance	252	1.73448e-4	6.03077e-3	3.0950	14	107	0.09%	5	12
	positive regulation of ion transport	254	1.84805e-4	6.37505e-3	2.0569	30	345	0.20%	22	74
	regulation of defense response to virus	261	2.22529e-4	7.47051e-3	2.2859	23	238	0.15%	15	54
	regulation of cartilage development	262	2.28094e-4	7.62808e-3	2.0600	29	333	0.19%	16	44
	peptide secretion	266	2.40529e-4	7.92300e-3	2.1504	26	286	0.17%	18	48
	positive regulation of developmental growth	275	2.83105e-4	9.02023e-3	2.2482	23	242	0.15%	17	39
	response to interleukin-6	277	3.03532e-4	9.60126e-3	4.0941	9	52	0.06%	7	13
	muscle cell fate determination	294	3.96946e-4	1.18301e-2	5.9137	6	24	0.04%	2	3
	regulation of synapse structure and activity	296	4.07177e-4	1.20530e-2	2.1504	24	264	0.16%	17	44
	regulation of response to biotic stimulus	300	4.46555e-4	1.30424e-2	2.0340	27	314	0.18%	19	69
	response to selenium ion	305	4.72865e-4	1.35844e-2	4.8701	7	34	0.05%	4	10
	response to electrical stimulus	307	5.26628e-4	1.50303e-2	2.5747	16	147	0.11%	14	31
	regulation of release of sequestered calcium ion into cytosol	312	5.72848e-4	1.60875e-2	2.7597	14	120	0.09%	8	25
	positive regulation of axon extension	314	5.77057e-4	1.61025e-2	2.6479	15	134	0.10%	10	23
	activation of protein kinase C activity by G-protein coupled receptor protein signaling pathway	316	5.88207e-4	1.63097e-2	2.4670	17	163	0.11%	10	38
	cellular component disassembly involved in apoptosis	317	6.09384e-4	1.68436e-2	2.0231	26	304	0.17%	19	72
	negative regulation of B cell proliferation	318	6.31417e-4	1.73977e-2	5.4588	6	26	0.04%	4	12
	histone H4 acetylation	322	6.74427e-4	1.83519e-2	2.6090	15	136	0.10%	10	33
	mammary placode formation	323	6.81452e-4	1.84857e-2	4.5995	7	36	0.05%	3	4
	muscle cell fate commitment	326	6.99645e-4	1.88046e-2	3.6705	9	58	0.06%	4	11
	interleukin-6-mediated signaling pathway	327	7.27653e-4	1.94975e-2	6.5707	5	18	0.03%	3	5
	cell aging	328	7.27719e-4	1.94399e-2	2.0644	24	275	0.16%	15	55
	retinal ganglion cell axon guidance	330	7.45735e-4	1.98004e-2	3.1349	11	83	0.07%	7	15
	regulation of neuronal synaptic plasticity	340	9.34529e-4	2.40834e-2	2.0275	24	280	0.16%	17	44
	synaptic vesicle docking involved in exocytosis	341	9.56908e-4	2.45878e-2	4.3574	7	38	0.05%	4	7
	central nervous system neuron development	344	9.95723e-4	2.53620e-2	2.1801	20	217	0.13%	11	41
	peripheral nervous system neuron development	347	1.00697e-3	2.54266e-2	3.0256	11	86	0.07%	3	10
	regulation of calcium ion transport into cytosol	356	1.12707e-3	2.77398e-2	2.2648	18	188	0.12%	11	40
	tight junction assembly	357	1.13249e-3	2.77951e-2	2.4813	15	143	0.10%	9	31
	monocyte chemotaxis	358	1.16858e-3	2.86008e-2	4.8941	6	29	0.04%	4	8
	ventricular cardiac muscle cell differentiation	364	1.21897e-3	2.93424e-2	3.1539	10	75	0.07%	7	17

	response to cholesterol	369	1.27371e-3	3.02445e-2	3.7105	8	51	0.05%	5	12
	transcription initiation from RNA polymerase II promoter	371	1.29986e-3	3.06990e-2	2.0488	22	254	0.15%	18	69
	positive regulation of calcium ion transport	371	1.29986e-3	3.06990e-2	2.0488	22	254	0.15%	16	57
	spleen development	377	1.36187e-3	3.16517e-2	2.5280	14	131	0.09%	11	24
	ectodermal placode formation	378	1.39289e-3	3.22872e-2	2.7559	12	103	0.08%	7	17
	retina morphogenesis in camera-type eye	381	1.40214e-3	3.22455e-2	2.3508	16	161	0.11%	12	31
	cellular response to lipid	384	1.46844e-3	3.35065e-2	2.8911	11	90	0.07%	7	21
	regulation of erythrocyte differentiation	385	1.47193e-3	3.34989e-2	2.2719	17	177	0.11%	12	29
	apical junction assembly	390	1.59450e-3	3.58232e-2	2.3219	16	163	0.11%	10	35
	regulation of amyloid precursor protein biosynthetic process	396	1.68497e-3	3.72820e-2	4.5783	6	31	0.04%	3	5
	histone ubiquitination	401	1.75734e-3	3.83985e-2	2.5626	13	120	0.09%	10	28
	positive regulation of small GTPase mediated signal transduction	401	1.75734e-3	3.83985e-2	2.5626	13	120	0.09%	9	28
	dendrite morphogenesis	403	1.76380e-3	3.83484e-2	2.2340	17	180	0.11%	11	27
	protein oxidation	405	1.78707e-3	3.86624e-2	23.6546	2	2	0.01%	1	1
	activation of phospholipase D activity by G-protein coupled receptor protein signaling pathway	405	1.78707e-3	3.86624e-2	23.6546	2	2	0.01%	1	1
	protein kinase C deactivation	405	1.78707e-3	3.86624e-2	23.6546	2	2	0.01%	1	1
	prepulse inhibition	408	1.79720e-3	3.85957e-2	3.2256	9	66	0.06%	5	13
	regulation of chondrocyte differentiation	409	1.80599e-3	3.86896e-2	2.0750	20	228	0.13%	9	31
	negative regulation of transporter activity	412	1.89389e-3	4.02774e-2	2.5414	13	121	0.09%	10	24
	negative regulation of ion transmembrane transporter activity	413	1.91772e-3	4.06853e-2	2.7979	11	93	0.07%	8	17
	fat-soluble vitamin metabolic process	416	1.92425e-3	4.05295e-2	2.2800	16	166	0.11%	14	45
	angiogenesis involved in wound healing	417	1.94215e-3	4.08085e-2	5.3760	5	22	0.03%	4	6
	cell differentiation involved in metanephros development	420	1.99925e-3	4.17081e-2	3.1775	9	67	0.06%	5	8
	distal tubule development	421	2.00028e-3	4.16306e-2	4.4352	6	32	0.04%	3	5
	renal system process	422	2.00143e-3	4.15557e-2	2.0569	20	230	0.13%	13	49
	cellular response to osmotic stress	423	2.03250e-3	4.21011e-2	3.8507	7	43	0.05%	5	7
	negative regulation of insulin secretion	432	2.22020e-3	4.50310e-2	2.3191	15	153	0.10%	10	27
	interleukin-12-mediated signaling pathway	434	2.27112e-3	4.58514e-2	6.7585	4	14	0.03%	2	3
	myoblast proliferation	438	2.32522e-3	4.65150e-2	10.1377	3	7	0.02%	2	4
	glutamate catabolic process	442	2.39588e-3	4.74948e-2	5.1423	5	23	0.03%	3	5
	negative regulation of cytokine biosynthetic process	446	2.45082e-3	4.81481e-2	2.2263	16	170	0.11%	11	24
GO Cellular Component	AP-2 adaptor complex	46	4.06640e-4	9.90963e-3	3.9424	9	54	0.06%	6	9
	clathrin coat of endocytic vesicle	55	9.00428e-4	1.83524e-2	3.5482	9	60	0.06%	6	10
	clathrin sculpted gamma-aminobutyric acid transport vesicle membrane	59	1.11481e-3	2.11814e-2	3.7847	8	50	0.05%	4	8
	inner mucus layer	63	1.55454e-3	2.76609e-2	5.6320	5	21	0.03%	1	1
	outer mucus layer	63	1.55454e-3	2.76609e-2	5.6320	5	21	0.03%	1	1
	SAGA-type complex	66	1.89389e-3	3.21675e-2	2.5414	13	121	0.09%	7	27
	clathrin coat of coated pit	70	2.21900e-3	3.55356e-2	3.1308	9	68	0.06%	6	14
	clathrin-coated endocytic vesicle membrane	72	2.45752e-3	3.82622e-2	3.0854	9	69	0.06%	6	14
Mouse Phenotype	abnormal aggression-related behavior	69	3.49871e-8	3.69849e-6	2.8012	36	304	0.24%	27	73
	weakness	73	9.97917e-8	9.97097e-6	2.1720	55	599	0.37%	35	112
	decreased aggression	112	1.28188e-6	8.34824e-5	3.3564	21	148	0.14%	14	41
	decreased aggression towards mice	116	1.52081e-6	9.56274e-5	4.1138	16	92	0.11%	10	24
	abnormal heart right ventricle size	122	2.18638e-6	1.30717e-4	2.1332	45	499	0.30%	32	90
	abnormal presacral vertebrae	127	2.64655e-6	1.52000e-4	4.1743	15	85	0.10%	3	5
	increased lumbar vertebrae number	131	2.84437e-6	1.58373e-4	4.7309	13	65	0.09%	2	5
	cervical vertebral transformation	135	3.57238e-6	1.93014e-4	2.4942	31	294	0.21%	21	45
	increased presacral vertebrae number	139	4.06086e-6	2.13093e-4	4.5897	13	67	0.09%	2	3
	abnormal palatal shelf elevation	148	6.70991e-6	3.30690e-4	2.5036	29	274	0.19%	16	39
	increased heart right ventricle size	158	1.07905e-5	4.98138e-4	2.1026	40	450	0.27%	28	80
	increased cell proliferation	173	1.77046e-5	7.46460e-4	2.0375	41	476	0.27%	24	86
	fused carpal bones	178	2.00179e-5	8.20285e-4	3.2430	17	124	0.11%	6	16
	abnormal circulating dihydrotestosterone level	188	2.84352e-5	1.10323e-3	11.8273	5	10	0.03%	3	3
	domed cranium	192	3.17143e-5	1.20481e-3	2.0691	37	423	0.25%	22	69
	abnormal pituitary gland development	193	3.33284e-5	1.25957e-3	2.5907	23	210	0.15%	15	30
	abnormal patella morphology	198	4.10366e-5	1.51172e-3	2.9568	18	144	0.12%	7	20
	scaly skin	201	4.45584e-5	1.61696e-3	2.4336	25	243	0.17%	17	54
	jerky movement	202	4.50658e-5	1.62728e-3	3.7049	13	83	0.09%	7	17
	micrognathia	209	5.44802e-5	1.90133e-3	2.4039	25	246	0.17%	17	34
	thin uterus	210	5.46214e-5	1.89718e-3	5.7344	8	33	0.05%	5	10

	abnormal neocortex morphology	211	5.48779e-5	1.89706e-3	3.0010	17	134	0.11%	9	25
	decreased circulating dihydrotestosterone level	227	7.55387e-5	2.42722e-3	23.6546	3	3	0.02%	1	1
	absent femur	228	7.78070e-5	2.48914e-3	6.3685	7	26	0.05%	4	4
	increased neurofibrosarcoma incidence	229	7.93255e-5	2.52664e-3	4.8384	9	44	0.06%	6	9
	absent sacral vertebrae	234	8.30296e-5	2.58811e-3	3.3116	14	100	0.09%	3	9
	decreased prepulse inhibition	241	9.47000e-5	2.86615e-3	2.4781	22	210	0.15%	12	40
	prostate gland hyperplasia	242	9.54443e-5	2.87674e-3	4.7309	9	45	0.06%	7	10
	intestinal adenocarcinoma	244	9.67834e-5	2.89319e-3	2.4180	23	225	0.15%	13	43
	abnormal branchial arch artery morphology	249	1.09888e-4	3.21896e-3	2.0625	32	367	0.21%	21	52
	decreased startle reflex	250	1.10047e-4	3.21072e-3	2.3010	25	257	0.17%	15	54
	pancreatic ductal adenocarcinoma	252	1.14220e-4	3.30603e-3	4.6281	9	46	0.06%	6	12
	small lung saccule	252	1.14220e-4	3.30603e-3	4.6281	9	46	0.06%	4	8
	abnormal neural plate morphology	255	1.17012e-4	3.34700e-3	2.2921	25	258	0.17%	16	35
	paraparesis	256	1.18244e-4	3.36902e-3	3.0588	15	116	0.10%	8	24
	decreased pancreatic acinar cell number	260	1.30606e-4	3.66400e-3	5.1145	8	37	0.05%	2	6
	muscle spasm	264	1.36185e-4	3.76263e-3	2.0933	30	339	0.20%	20	61
	muscle twitch	264	1.36185e-4	3.76263e-3	2.0933	30	339	0.20%	20	62
	ascites	271	1.56114e-4	4.20183e-3	2.5164	20	188	0.13%	14	31
	abnormal prepulse inhibition	272	1.58718e-4	4.25620e-3	2.1365	28	310	0.19%	17	63
	increased fibroma incidence	273	1.61113e-4	4.30460e-3	4.4352	9	48	0.06%	6	11
	abnormal vertebral transverse process morphology	277	1.73448e-4	4.56727e-3	3.0950	14	107	0.09%	5	7
	abnormal secondary ovarian follicle morphology	281	1.90339e-4	4.94069e-3	2.9324	15	121	0.10%	9	29
	thin cerebral cortex	291	2.31017e-4	5.79052e-3	2.2351	24	254	0.16%	15	65
	absent patella	295	2.54205e-4	6.28533e-3	3.1379	13	98	0.09%	4	7
	abnormal liver development	296	2.54422e-4	6.26944e-3	2.0763	28	319	0.19%	21	55
	abnormal rib development	300	2.59553e-4	6.31061e-3	2.2176	24	256	0.16%	15	30
	distended pericardium	301	2.65088e-4	6.42376e-3	2.1741	25	272	0.17%	20	59
	abnormal renal glomerulus basement membrane morphology	304	2.81463e-4	6.75325e-3	2.9568	14	112	0.09%	8	13
	abnormal pancreatic acinar cell morphology	305	2.86314e-4	6.84712e-3	2.4694	19	182	0.13%	11	38
	abnormal ilium morphology	308	3.11046e-4	7.36613e-3	3.0751	13	100	0.09%	4	9
	decreased aggression towards males	313	3.30789e-4	7.70855e-3	4.5056	8	42	0.05%	6	13
	dermal cysts	314	3.34650e-4	7.77368e-3	3.2256	12	88	0.08%	5	9
	enlarged prostate gland	317	3.51985e-4	8.09898e-3	4.0168	9	53	0.06%	7	16
	decreased tongue size	322	3.78371e-4	8.57092e-3	3.0148	13	102	0.09%	8	15
	pale liver	323	3.79283e-4	8.56499e-3	2.0255	28	327	0.19%	23	75
	increased melanoma incidence	325	3.91414e-4	8.78454e-3	4.4009	8	43	0.05%	5	10
	diarrhea	327	4.03110e-4	8.99170e-3	2.4034	19	187	0.13%	13	41
	increased pancreatic islet number	328	4.06640e-4	9.04277e-3	3.9424	9	54	0.06%	3	9
	short maxilla	329	4.07177e-4	9.02720e-3	2.1504	24	264	0.16%	16	42
	abnormal pancreatic islet number	334	4.17761e-4	9.12320e-3	2.7294	15	130	0.10%	7	21
	myoclonus	338	4.30879e-4	9.29831e-3	2.3906	19	188	0.13%	11	31
	retinal ganglion cell degeneration	339	4.37649e-4	9.41655e-3	3.5840	10	66	0.07%	6	15
	abnormal Rathke's pouch	343	4.55211e-4	9.68021e-3	2.4470	18	174	0.12%	11	25
	abnormal bronchus epithelium morphology	344	4.57531e-4	9.70126e-3	3.1193	12	91	0.08%	8	16
	abnormal rhombomere morphology	346	4.58474e-4	9.66506e-3	2.3191	20	204	0.13%	13	25
	increased spindle cell carcinoma incidence	347	4.72865e-4	9.93970e-3	4.8701	7	34	0.05%	4	6
	abnormal pulmonary respiratory rate	349	4.79120e-4	1.00135e-2	2.2579	21	220	0.14%	16	44
	abnormal CNS synapse formation	351	4.88236e-4	1.01458e-2	2.5923	16	146	0.11%	8	26
	squamous cell carcinoma	359	5.39685e-4	1.09651e-2	2.2376	21	222	0.14%	10	45
	abnormal neuromere morphology	369	5.87223e-4	1.16076e-2	2.2745	20	208	0.13%	13	26
	increased retinal ganglion cell number	372	6.13911e-4	1.20373e-2	3.7349	9	57	0.06%	4	10
	increased mesothelioma incidence	379	6.81452e-4	1.31148e-2	4.5995	7	36	0.05%	4	8
	increased cholangiocarcinoma incidence	379	6.81452e-4	1.31148e-2	4.5995	7	36	0.05%	5	6
	decreased salivation	384	7.06100e-4	1.34122e-2	3.3792	10	70	0.07%	6	17
	abnormal orientation of cochlear hair cell stereociliary bundles	392	7.64923e-4	1.42330e-2	2.2814	19	197	0.13%	11	36
	decreased circulating estrogen level	393	7.85416e-4	1.45772e-2	2.5712	15	138	0.10%	9	28
	ventricular hypoplasia	397	8.15635e-4	1.49855e-2	2.4737	16	153	0.11%	9	29
	decreased renal tubule number	399	8.25689e-4	1.50942e-2	3.0976	11	84	0.07%	4	6
	small limb buds	399	8.25689e-4	1.50942e-2	3.0976	11	84	0.07%	7	12
	abnormal macrophage activation involved in immune response	403	8.44429e-4	1.52835e-2	3.9424	8	48	0.05%	4	7
	abnormal extraembryonic endoderm formation	406	8.80208e-4	1.58134e-2	2.3140	18	184	0.12%	10	35
	abnormal pancreatic acinus morphology	408	8.88418e-4	1.58826e-2	2.2004	20	215	0.13%	12	44
	abnormal pulmonary alveolar duct morphology	409	9.00428e-4	1.60580e-2	3.5482	9	60	0.06%	4	14
	absent amacrine cells	415	9.60969e-4	1.68899e-2	5.0688	6	28	0.04%	1	4
	abnormal susceptibility to hearing loss	420	9.97143e-4	1.73170e-2	2.2892	18	186	0.12%	13	34

	abnormal prostate gland size	422	1.00472e-3	1.73660e-2	2.4261	16	156	0.11%	13	37
	abnormal heart shape	426	1.05430e-3	1.80518e-2	2.4987	15	142	0.10%	12	29
	abnormal pancreas development	429	1.06177e-3	1.80525e-2	2.1228	21	234	0.14%	12	32
	increased hepatocyte apoptosis	432	1.08158e-3	1.82617e-2	2.0060	24	283	0.16%	18	56
	pancreatic intraepithelial neoplasia	437	1.14561e-3	1.91214e-2	3.4337	9	62	0.06%	6	11
	abnormal primary somatosensory cortex morphology	438	1.17316e-3	1.95366e-2	2.8104	12	101	0.08%	9	18
	increased vertebrae number	439	1.17358e-3	1.94990e-2	2.5672	14	129	0.09%	3	13
	abnormal conotruncus morphology	441	1.18148e-3	1.95414e-2	2.1049	21	236	0.14%	11	33
	failure of palatal shelf elevation	443	1.19726e-3	1.97128e-2	2.2528	18	189	0.12%	10	29
	cleft chin	447	1.20199e-3	1.96136e-2	7.8849	4	12	0.03%	2	3
	abnormal circulating potassium level	448	1.21550e-3	1.97898e-2	2.4640	15	144	0.10%	9	29
	increased aggression	449	1.22190e-3	1.98498e-2	2.3111	17	174	0.11%	15	36
	abnormal alveolocapillary membrane morphology	450	1.22691e-3	1.98869e-2	5.9137	5	20	0.03%	2	3
	abnormal serous gland morphology	450	1.22691e-3	1.98869e-2	5.9137	5	20	0.03%	3	3
	mammary gland hyperplasia	454	1.27913e-3	2.05506e-2	2.7829	12	102	0.08%	8	18
	arteritis	455	1.31314e-3	2.10507e-2	4.1396	7	40	0.05%	5	13
	enlarged lung	460	1.37642e-3	2.18253e-2	2.1712	19	207	0.13%	11	48
	short sternum	461	1.38266e-3	2.18766e-2	2.0784	21	239	0.14%	13	39
	increased grooming behavior	463	1.39289e-3	2.19434e-2	2.7559	12	103	0.08%	7	16
	increased glioblastoma incidence	464	1.40884e-3	2.21467e-2	4.7309	6	30	0.04%	4	7
	patent ductus arteriosus	466	1.44206e-3	2.25716e-2	3.3264	9	64	0.06%	6	16
	abnormal cortical ventricular zone morphology	466	1.44206e-3	2.25716e-2	3.3264	9	64	0.06%	5	10
	absent ulna	471	1.49474e-3	2.31479e-2	3.0720	10	77	0.07%	3	8
	abnormal cricoid cartilage morphology	472	1.49573e-3	2.31140e-2	2.3363	16	162	0.11%	9	17
	increased keratinocyte proliferation	472	1.49573e-3	2.31140e-2	2.3363	16	162	0.11%	9	31
	enlarged hair follicles	479	1.55454e-3	2.36718e-2	5.6320	5	21	0.03%	2	4
	progressive muscle weakness	485	1.60744e-3	2.41746e-2	2.8593	11	91	0.07%	9	29
	vasculitis	485	1.60744e-3	2.41746e-2	2.8593	11	91	0.07%	10	25
	muscular ventricular septal defect	488	1.62902e-3	2.43485e-2	2.5841	13	119	0.09%	7	18
	kidney medulla hypoplasia	490	1.64552e-3	2.44948e-2	2.7034	12	105	0.08%	5	8
	abnormal hippocampal fimbria	491	1.65047e-3	2.45184e-2	3.0326	10	78	0.07%	6	10
	impaired branching involved in terminal bronchiole morphogenesis	491	1.65047e-3	2.45184e-2	3.0326	10	78	0.07%	5	11
	small ischium	497	1.67820e-3	2.46294e-2	7.2783	4	13	0.03%	1	2
	absent awl hair	497	1.67820e-3	2.46294e-2	7.2783	4	13	0.03%	1	1
	delayed male fertility	497	1.67820e-3	2.46294e-2	7.2783	4	13	0.03%	3	6
	absent auchene hairs	500	1.68497e-3	2.45803e-2	4.5783	6	31	0.04%	2	5
	L5 dorsal root ganglion hypertrophy	504	1.78707e-3	2.58628e-2	23.6546	2	2	0.01%	1	1
	enlarged prostate gland anterior lobe	504	1.78707e-3	2.58628e-2	23.6546	2	2	0.01%	2	2
	abnormal autophagy	512	1.91772e-3	2.73200e-2	2.7979	11	93	0.07%	6	22
	increased susceptibility to noise-induced hearing loss	515	1.99925e-3	2.83155e-2	3.1775	9	67	0.06%	6	13
	uterus adenomyosis	517	2.03250e-3	2.86752e-2	3.8507	7	43	0.05%	4	6
	increased neurofibroma incidence	517	2.03250e-3	2.86752e-2	3.8507	7	43	0.05%	5	8
	increased pulmonary respiratory rate	519	2.03907e-3	2.86570e-2	2.5206	13	122	0.09%	10	25
	abnormal intestinal goblet cells	520	2.06734e-3	2.89984e-2	2.0111	21	247	0.14%	12	43
	abnormal sternum ossification	520	2.06734e-3	2.89984e-2	2.0111	21	247	0.14%	13	39
	abnormal oviduct morphology	523	2.09018e-3	2.91507e-2	2.7681	11	94	0.07%	8	18
	increased prostate gland tumor incidence	523	2.09018e-3	2.91507e-2	2.7681	11	94	0.07%	6	26
	absent ureter	530	2.19329e-3	3.01846e-2	2.5001	13	123	0.09%	7	18
	abnormal orientation of outer hair cell stereociliary bundles	530	2.19329e-3	3.01846e-2	2.5001	13	123	0.09%	8	24
	reduced linear vestibular evoked potential	532	2.19798e-3	3.01355e-2	2.9203	10	81	0.07%	5	13
	pancreas cysts	537	2.27502e-3	3.09014e-2	2.7390	11	95	0.07%	7	22
	increased cochlear nerve compound action potential	540	2.32522e-3	3.14077e-2	10.1377	3	7	0.02%	2	2
	abnormal memory B cell morphology	541	2.32986e-3	3.14121e-2	3.7632	7	44	0.05%	5	14
	small pubis	541	2.32986e-3	3.14121e-2	3.7632	7	44	0.05%	4	7
	social withdrawal	543	2.35793e-3	3.16736e-2	3.3792	8	56	0.05%	7	8
	abnormal visual cortex morphology	544	2.35819e-3	3.16189e-2	4.3008	6	33	0.04%	3	7
	absent retinal inner plexiform layer	544	2.35819e-3	3.16189e-2	4.3008	6	33	0.04%	1	4
	skeletal muscle fibrosis	547	2.38561e-3	3.18110e-2	2.3825	14	139	0.09%	10	26
	abnormal auditory cortex morphology	548	2.39588e-3	3.18897e-2	5.1423	5	23	0.03%	2	4
	absent ilium	548	2.39588e-3	3.18897e-2	5.1423	5	23	0.03%	2	2
	abnormal extraocular muscle morphology	548	2.39588e-3	3.18897e-2	5.1423	5	23	0.03%	4	5
	necrospermia	548	2.39588e-3	3.18897e-2	5.1423	5	23	0.03%	2	5
	hyperpigmentation	552	2.44524e-3	3.23109e-2	2.5805	12	110	0.08%	8	27
	decreased synaptic depression	555	2.45752e-3	3.22976e-2	3.0854	9	69	0.06%	5	10
	abnormal second branchial arch morphology	561	2.63810e-3	3.43001e-2	2.1504	17	187	0.11%	13	37
	prostate gland adenocarcinoma	563	2.64267e-3	3.42374e-2	3.3199	8	57	0.05%	5	15

	abnormal pancreatic beta cell differentiation	565	2.65961e-3	3.43349e-2	3.6796	7	45	0.05%	2	7
	abnormal nest building behavior	567	2.68441e-3	3.45328e-2	2.6825	11	97	0.07%	8	18
	abnormal anterior visceral endoderm morphology	567	2.68441e-3	3.45328e-2	2.6825	11	97	0.07%	5	19
	abnormal accessory nerve morphology	571	2.76219e-3	3.52845e-2	4.1743	6	34	0.04%	3	4
	bronchiolar epithelial hyperplasia	571	2.76219e-3	3.52845e-2	4.1743	6	34	0.04%	4	5
	abnormal vestibulocochlear nerve morphology	574	2.84288e-3	3.61254e-2	2.5344	12	112	0.08%	8	19
	small vestibular ganglion	577	2.88273e-3	3.64413e-2	2.8160	10	84	0.07%	6	14
	abnormal pancreatic alpha cell morphology	578	2.89454e-3	3.65272e-2	2.0337	19	221	0.13%	12	35
	abnormal barrel cortex morphology	579	2.91029e-3	3.66627e-2	2.6551	11	98	0.07%	8	17
	proctitis	580	2.92191e-3	3.67456e-2	4.9280	5	24	0.03%	3	3
	increased grip strength	580	2.92191e-3	3.67456e-2	4.9280	5	24	0.03%	3	6
	increased stomach tumor incidence	589	3.15121e-3	3.90237e-2	2.6283	11	99	0.07%	8	22
	decreased circulating follicle stimulating hormone level	593	3.21411e-3	3.95340e-2	2.2316	15	159	0.10%	9	30
	abnormal amacrine cell morphology	595	3.27208e-3	4.01119e-2	2.1627	16	175	0.11%	9	35
	decreased follicle stimulating hormone level	596	3.28220e-3	4.01684e-2	2.1054	17	191	0.11%	11	38
	abnormal cochlear nerve morphology	598	3.29217e-3	4.01557e-2	3.2074	8	59	0.05%	5	12
	tachypnea	599	3.29694e-3	4.01467e-2	2.9568	9	72	0.06%	7	14
	abnormal penis morphology	600	3.29974e-3	4.01138e-2	2.2998	14	144	0.09%	9	25
	pale placenta	604	3.40788e-3	4.11541e-2	2.6020	11	100	0.07%	8	11
	fusion of glossopharyngeal and vagus nerve	604	3.40788e-3	4.11541e-2	2.6020	11	100	0.07%	6	12
	abnormal parietal lobe morphology	608	3.46087e-3	4.15191e-2	2.1504	16	176	0.11%	13	33
	decreased circulating sodium level	611	3.52648e-3	4.20984e-2	4.7309	5	25	0.03%	3	9
	abnormal interventricular septum muscular part morphology	613	3.56216e-3	4.23856e-2	2.3655	13	130	0.09%	7	20
	decreased circulating antidiuretic hormone level	616	3.60345e-3	4.26682e-2	8.8705	3	8	0.02%	2	2
	impaired synaptic plasticity	618	3.65002e-3	4.30797e-2	2.0836	17	193	0.11%	12	31
	abnormal sesamoid bone of gastrocnemius morphology	619	3.66013e-3	4.31292e-2	3.1539	8	60	0.05%	2	3
	abnormal endplate potential	624	3.79536e-3	4.43644e-2	2.4470	12	116	0.08%	8	15
	absent ureteric bud	625	3.80456e-3	4.44007e-2	2.3474	13	131	0.09%	6	12
	abnormal vagina opening	627	3.84630e-3	4.47446e-2	2.0728	17	194	0.11%	12	39
	failure of atrioventricular cushion closure	629	3.86602e-3	4.48311e-2	3.4496	7	48	0.05%	4	9
	constricted vagina opening	629	3.86602e-3	4.48311e-2	3.4496	7	48	0.05%	3	6
	vascular inflammation	634	3.97135e-3	4.56893e-2	2.5510	11	102	0.07%	10	30
	darkened coat color	634	3.97135e-3	4.56893e-2	2.5510	11	102	0.07%	6	18
	decreased pulmonary ventilation	639	4.04930e-3	4.62216e-2	2.6880	10	88	0.07%	6	13
	abnormal branching involved in terminal bronchiole morphogenesis	639	4.04930e-3	4.62216e-2	2.6880	10	88	0.07%	5	13
	abnormal vascular regression	643	4.08169e-3	4.63015e-2	2.1144	16	179	0.11%	12	39
	abnormal fetal ductus arteriosus morphology	649	4.22140e-3	4.74436e-2	2.2376	14	148	0.09%	10	25
	abnormal ureter development	652	4.26493e-3	4.77123e-2	2.0517	17	196	0.11%	9	23
	abnormal occipital lobe morphology	653	4.28646e-3	4.78797e-2	3.8359	6	37	0.04%	3	9
	absent branchial arches	656	4.34676e-3	4.83312e-2	2.8386	9	75	0.06%	8	9
	increased fibrosarcoma incidence	656	4.34676e-3	4.83312e-2	2.8386	9	75	0.06%	6	18
	abnormal hair follicle outer root sheath morphology	660	4.39232e-3	4.85418e-2	2.6578	10	89	0.07%	8	16
	hyperresponsive to tactile stimuli	664	4.49071e-3	4.93302e-2	3.0522	8	62	0.05%	5	10
	abnormal conotruncus septation	664	4.49071e-3	4.93302e-2	3.0522	8	62	0.05%	4	6
Disease Ontology	Conn syndrome	44	5.07396e-6	2.57619e-4	9.1990	7	18	0.05%	5	8
	middle ear disease	56	9.52958e-6	3.80162e-4	2.7294	24	208	0.16%	17	51
	primary Escherichia coli infectious disease	68	1.94168e-5	6.37899e-4	3.7632	14	88	0.09%	3	9
	commensal Escherichia coli infectious disease	68	1.94168e-5	6.37899e-4	3.7632	14	88	0.09%	3	9
	primary Enterobacteriaceae infectious disease	79	4.52652e-5	1.28003e-3	3.0464	17	132	0.11%	5	16
	myopia	83	5.33988e-5	1.43726e-3	2.7189	20	174	0.13%	14	40
	otitis media	89	8.40485e-5	2.10971e-3	3.4944	13	88	0.09%	7	23
	refractive error	94	1.88447e-4	4.47864e-3	2.2232	25	266	0.17%	19	54
	Hashimoto Disease	110	5.67576e-4	1.15270e-2	2.5573	16	148	0.11%	11	41
	cervical adenocarcinoma	127	9.12613e-4	1.60534e-2	3.0612	11	85	0.07%	7	19
	glandular cystitis	134	1.11481e-3	1.85857e-2	3.7847	8	50	0.05%	3	6
	mucinous adenocarcinoma	135	1.14561e-3	1.89577e-2	3.4337	9	62	0.06%	3	9
	Hirschsprung's disease	137	1.39556e-3	2.27567e-2	2.6283	13	117	0.09%	9	17
	actinic keratosis	139	1.44206e-3	2.31766e-2	3.3264	9	64	0.06%	6	19
	Burkitt's lymphoma	140	1.56432e-3	2.49621e-2	2.2591	17	178	0.11%	14	39
	endemic goiter	145	1.78707e-3	2.75332e-2	23.6546	2	2	0.01%	1	1
	background diabetic retinopathy	145	1.78707e-3	2.75332e-2	23.6546	2	2	0.01%	1	1
	chronic cystitis	147	1.81908e-3	2.76450e-2	2.9943	10	79	0.07%	5	15
	Charcot-Marie-Tooth disease	149	1.87129e-3	2.80567e-2	2.2217	17	181	0.11%	9	31
	megacolon	150	1.89389e-3	2.82064e-2	2.5414	13	121	0.09%	9	19

MSigDB Cancer Neighborhood	Neighborhood of CASP10	2	4.07057e-6	8.69066e-4	2.1026	44	495	0.29%	34	109
	Neighborhood of SMO	15	1.00583e-3	2.86326e-2	2.0900	22	249	0.15%	16	51
	Neighborhood of DDX11	16	1.12707e-3	3.00787e-2	2.2648	18	188	0.12%	13	38
	Neighborhood of AQP4	17	1.22872e-3	3.08626e-2	2.1924	19	205	0.13%	14	43
	Neighborhood of RTN1	18	1.66154e-3	3.94155e-2	2.2465	17	179	0.11%	11	44
	Neighborhood of JUND	20	1.98422e-3	4.23630e-2	2.2095	17	182	0.11%	15	67
PANTHER Pathway	TGF-beta signaling pathway	1	3.26658e-6	4.57321e-4	2.0458	48	555	0.32%	32	95
	General transcription regulation	2	8.75483e-5	6.12838e-3	3.1400	15	113	0.10%	12	35
	Ras Pathway	3	1.38449e-4	6.46098e-3	2.0119	33	388	0.22%	22	70
	Beta2 adrenergic receptor signaling pathway	14	2.56960e-3	2.56959e-2	2.0132	20	235	0.13%	15	43
	Gamma-aminobutyric acid synthesis	17	3.52648e-3	2.90416e-2	4.7309	5	25	0.03%	3	5
	Transcription regulation by bZIP transcription factor	19	4.72024e-3	3.47807e-2	2.0310	17	198	0.11%	14	51
Pathway Commons	NCAM signaling for neurite out-growth	3	1.05641e-5	5.65883e-3	2.2316	35	371	0.23%	23	57
	Cell junction organization	22	8.81267e-5	6.43726e-3	2.0361	34	395	0.23%	23	70
	N-Glycan antennae elongation	50	1.35988e-4	4.37065e-3	4.5296	9	47	0.06%	4	9
	Signaling by Interleukins	58	3.38000e-4	9.36494e-3	2.0117	29	341	0.19%	26	93
	Tight junction interactions	61	5.43390e-4	1.43152e-2	3.2525	11	80	0.07%	6	19
	NCAM1 interactions	63	5.96006e-4	1.52029e-2	2.3287	19	193	0.13%	10	23
	Stimulation of the cell death response by PAK-2p34	68	7.27653e-4	1.71962e-2	6.5707	5	18	0.03%	3	3
	Cell-cell junction organization	70	9.52608e-4	2.18692e-2	2.1411	21	232	0.14%	15	46
	Voltage gated Potassium channels	72	1.00583e-3	2.24496e-2	2.0900	22	249	0.15%	14	40
	Transport to the Golgi and subsequent modification	73	1.01129e-3	2.22622e-2	2.7213	13	113	0.09%	7	28
	N-glycan antennae elongation in the medial/trans-Golgi	74	1.01705e-3	2.20865e-2	3.4900	9	61	0.06%	4	12
	CDO in myogenesis	75	1.12707e-3	2.41493e-2	2.2648	18	188	0.12%	12	24
	Myogenesis	75	1.12707e-3	2.41493e-2	2.2648	18	188	0.12%	12	24
	Transcription of the HIV genome	77	1.22872e-3	2.56435e-2	2.1924	19	205	0.13%	14	61
	Ras signaling in the CD4+ TCR pathway	78	1.30079e-3	2.67996e-2	2.1817	19	206	0.13%	14	42
	Regulation of PAK-2p34 activity by PS-GAP/RHG10	79	1.37190e-3	2.79069e-2	11.8273	3	6	0.02%	1	2
	p38MAPK events	81	1.64552e-3	3.26464e-2	2.7034	12	105	0.08%	7	9
	CD28 dependent Vav1 pathway	82	1.75701e-3	3.44330e-2	2.8283	11	92	0.07%	7	18
	Hormone ligand-binding receptors	84	2.00028e-3	3.82673e-2	4.4352	6	32	0.04%	3	10
	Signalling to RAS	85	2.04608e-3	3.86830e-2	2.2663	16	167	0.11%	11	21
MSigDB Pathway	Arrhythmogenic right ventricular cardiomyopathy (ARVC)	1	1.21032e-6	1.06508e-3	2.3022	40	411	0.27%	32	74
	Renal cell carcinoma	2	4.88970e-6	2.15147e-3	2.3448	34	343	0.23%	25	70
	Genes related to PIP3 signaling in B lymphocytes	5	5.42234e-5	9.54331e-3	3.1279	16	121	0.11%	13	34
	Agrin in Postsynaptic Differentiation	7	1.09022e-4	1.37056e-2	2.6594	19	169	0.13%	12	36
	Genes involved in MyoGenessis	11	2.39821e-4	1.91857e-2	2.3232	22	224	0.15%	15	29
	Fc Epsilon Receptor I Signaling in Mast Cells	12	2.72454e-4	1.99800e-2	2.4137	20	196	0.13%	14	38
	Angiotensin II mediated activation of JNK Pathway via Pyk2 dependent signaling	15	4.91425e-4	2.88303e-2	2.3655	19	190	0.13%	13	33
	Genes involved in Signaling by EGFR	18	5.22447e-4	2.55418e-2	2.1958	22	237	0.15%	17	49
	Genes involved in Signalling to RAS	23	8.06999e-4	3.08765e-2	2.1692	21	229	0.14%	13	26
	Links between Pyk2 and Map Kinases	25	9.45191e-4	3.32707e-2	2.3655	17	170	0.11%	12	28
	Genes related to chemotaxis	25	9.45191e-4	3.32707e-2	2.3655	17	170	0.11%	14	45
	FAS signaling pathway (CD95)	28	1.27913e-3	4.02013e-2	2.7829	12	102	0.08%	8	30
	Genes involved in Signalling to ERKs	30	1.66461e-3	4.88285e-2	2.0093	22	259	0.15%	14	34
	p38 MAPK Signaling Pathway	31	1.70112e-3	4.82899e-2	2.1835	18	195	0.12%	14	41
	Genes involved in Activation of Rac	32	1.79720e-3	4.94229e-2	3.2256	9	66	0.06%	6	14
	JNK MAPK Pathway	33	1.87129e-3	4.99010e-2	2.2217	17	181	0.11%	13	38
MGI Expression: Detected	TS9_extraembryonic component	254	3.23539e-8	1.06335e-6	2.0761	66	752	0.44%	44	133
	TS23_lens	260	3.98160e-8	1.27840e-6	2.2151	56	598	0.37%	33	122
	TS28_cerebral cortex layer; layer VI	306	2.68037e-7	7.31234e-6	2.5442	37	344	0.25%	20	54
	TS28_tectum	314	3.62226e-7	9.63012e-6	2.2794	45	467	0.30%	25	68
	TS28_cerebral cortex layer; layer III	321	4.59460e-7	1.19488e-5	2.1746	49	533	0.33%	30	97
	TS28_cerebral cortex layer; layer II	339	1.27556e-6	3.14111e-5	2.1768	45	489	0.30%	27	80
	TS17_tail; nervous system; central nervous system; future spinal cord; neural tube	346	1.63082e-6	3.93470e-5	3.0197	24	188	0.16%	10	19
	TS17_optic cup	357	2.31149e-6	5.40514e-5	2.0066	52	613	0.35%	35	90
	TS28_cerebral cortex layer; layer V	369	3.10165e-6	7.01696e-5	2.2159	40	427	0.27%	23	78
	TS28_cerebral cortex layer; layer IV	378	3.68109e-6	8.12956e-5	2.4497	32	309	0.21%	20	56
	TS21_arm	392	5.29916e-6	1.12850e-4	2.8244	24	201	0.16%	12	26
	TS17_tail; nervous system; central nervous system	397	5.77378e-6	1.21409e-4	2.8104	24	202	0.16%	10	25

	TS17_tail; nervous system; central nervous system; future spinal cord	397	5.77378e-6	1.21409e-4	2.8104	24	202	0.16%	10	24
	TS17_tail; nervous system	400	6.28676e-6	1.31205e-4	2.7966	24	203	0.16%	10	26
	TS25_cerebral cortex; intermediate zone	406	7.29506e-6	1.49998e-4	3.8567	15	92	0.10%	7	15
	TS20_pons	409	7.59796e-6	1.55080e-4	2.7003	25	219	0.17%	17	34
	TS21_neural retinal epithelium	414	8.65362e-6	1.74494e-4	2.2234	36	383	0.24%	24	52
	TS15_tail; mesenchyme	415	9.19265e-6	1.84916e-4	2.0002	46	544	0.31%	29	87
	TS18_pancreas primordium	421	1.14689e-5	2.27417e-4	8.2791	7	20	0.05%	2	4
	TS17_atrio-ventricular canal	429	1.27079e-5	2.47285e-4	4.1555	13	74	0.09%	7	12
MSigDB Perturbation	Genes down-regulated and displaying decreased copy number in glioblastoma samples.	9	1.45423e-7	3.83594e-5	4.8522	16	78	0.11%	10	29
	Genes down-regulated in Caco-2 cells (intestinal epithelium) after coculture with the probiotic bacteria L. casei for 6h.	15	6.81669e-7	1.07885e-4	2.0919	52	588	0.35%	38	173
	Selected gradually up-regulated genes whose expression profile follows that of HEMGN [Gene ID=55363] in the TLX1 [Gene ID=3195] Tet On iEBHX15-4 cells (pro-erythroblasts).	26	8.15062e-6	7.44215e-4	2.9924	21	166	0.14%	10	30
	Genes from the blue module which are up-regulated in HAEC cells (primary aortic endothelium) after exposure to the oxidized 1-palmitoyl-2-arachidonyl-sn-3-glycerophosphorylcholine (oxPAPC).	30	1.19598e-5	9.46420e-4	2.0933	40	452	0.27%	33	117
	Genes from the turquoise module which are up-regulated in HAEC cells (primary aortic endothelium) after exposure to the oxidized 1-palmitoyl-2-arachidonyl-sn-3-glycerophosphocholine (oxPAPC).	34	1.92442e-5	1.34370e-3	2.1974	34	366	0.23%	27	72
	Selected gradually up-regulated genes whose expression profile follows that of HBZ [Gene ID=3050] in the TLX1 [Gene ID=3195] Tet On iEBHX15-4 cells (pro-erythroblasts).	37	2.25585e-5	1.44740e-3	2.5923	24	219	0.16%	13	39
	Genes up-regulated in metaplastic carcinoma of the breast (MCB) subclass 2 compared to the MCB subclass 1.	43	4.56966e-5	2.52288e-3	2.6020	22	200	0.15%	16	33
	Genes consistently up-regulated in HMEC cells (primary mammary epithelium) upon expression of TERT [Gene ID=7015] off a retroviral vector.	53	1.01940e-4	4.56615e-3	2.0207	34	398	0.23%	26	131
	Genes commonly up-regulated in CD-1 and CD-2 clusters of multiple myeloma samples and which were higher expressed in the CD-1 group.	64	1.95376e-4	7.24724e-3	2.1432	27	298	0.18%	21	77
	Genes up-regulated in HCT8/S11 cells (colon cancer) engineered to stably express NTN1 [Gene ID=1630] off a plasmid vector.	65	2.00486e-4	7.32238e-3	2.8035	16	135	0.11%	6	18
	Down-regulated in ovarian epithelial cells (MCV152) 72 hours following FSH treatment, compared to untreated	72	2.72828e-4	8.99574e-3	2.8386	15	125	0.10%	11	47
	Genes down-regulated in poorly differentiated thyroid carcinoma (PDTC) compared to anaplastic thyroid carcinoma (ATC).	78	3.61178e-4	1.09928e-2	2.0317	28	326	0.19%	20	69
	Genes up-regulated in metastatic breast cancer tumors having type 2 amplification in the 20q13 region; involves MYBL2, STK6 and ZNF217 [Gene ID=4605, 6790, 7764]	81	3.98184e-4	1.16702e-2	2.0193	28	328	0.19%	20	106
	Genes up-regulated in MCF10A cells (breast cancer) by expression of LSM1 [Gene ID=27257] off a letiviral vector.	83	4.58474e-4	1.31135e-2	2.3191	20	204	0.13%	13	40
	Genes whose expression peaked at 60 min after stimulation of MCF10A cells with EGF [Gene ID=1950].	89	5.10502e-4	1.36172e-2	2.4977	17	161	0.11%	12	36
	Genes that cooperate with MYC [Gene ID=4609] and TBX2 [Gene ID=6909] to transform MEF cells (embryo fibroblasts).	96	7.45735e-4	1.84414e-2	3.1349	11	83	0.07%	7	11
	Genes down-regulated in A549 cells (lung cancer) expressing MIRLET7A3 [Gene ID=406883] microRNA off a plasmid vector.	111	1.34133e-3	2.86876e-2	2.0076	23	271	0.15%	19	77
	Genes up-regulated in MCF-7 cells (breast cancer) by selective estrogen receptor modulators (SERM) 4-hydroxytamoxifen, raloxifene, or ICI 182780 but not by estradiol [PubChem=44959, 5035, 3478439, 5757].	120	1.64532e-3	3.25500e-2	3.5705	8	53	0.05%	7	19
	Amplification hot spot 25: colocalized fragile sites and cancer genes in the 2q13-q36 region.	122	2.00132e-3	3.89437e-2	2.9568	10	80	0.07%	5	9
	Top genes up-regulated in subcutaneous tumors from highly metastatic pancreatic cancer cells.	126	2.32791e-3	4.38609e-2	2.0304	20	233	0.13%	11	42
MSigDB Predicted Promoter Motifs	Motif DGATADGAHWAGATA matches EVI1: ecotropic viral integration site 1	7	5.04266e-12	4.43034e-10	2.3594	78	782	0.52%	54	186
	Motif NNAACAATNN matches SOX5: SRY (sex determining region Y)-box 5	9	9.85693e-12	6.73557e-10	2.2477	84	884	0.56%	57	191

	Motif YNGTTNNNATT (no known TF)	10	1.43755e-11	8.84090e-10	2.0614	101	1159	0.67%	66	272
	Motif TGATTTRY matches GFI1: growth factor independent 1	11	1.54359e-10	8.63005e-9	2.0957	87	982	0.58%	63	233
	Motif NNNNNGTAAATAAACA matches FOXC1: forkhead box C1	14	2.08805e-10	9.17251e-9	2.0929	86	972	0.57%	61	191
	Motif NNNNAACAATRGNN matches SOX9: SRY (sex determining region Y)-box 9 (campomelic dysplasia, autosomal sex-reversal)	17	4.17455e-10	1.51021e-8	2.1738	76	827	0.51%	56	181
	Motif NAWTGTTTRTTT matches FOXD3: forkhead box D3	20	2.29658e-9	7.06199e-8	2.1396	72	796	0.48%	51	164
	Motif NNNNNNNWATGCAAATNNNWNNA matches POU2F1: POU domain, class 2, transcription factor 1	22	5.16741e-9	1.44453e-7	2.1363	69	764	0.46%	50	192
	Motif NNNTGTTTATNTR matches FOXJ1: forkhead box J1	24	8.15527e-9	2.08979e-7	2.0521	74	853	0.49%	44	170
	Motif KGNANTRTTTRYTTW matches FOXA2: forkhead box A2	29	2.98044e-8	6.32058e-7	2.0317	70	815	0.47%	42	170
	Motif CAGNYGKNAAA (no known TF)	31	4.56150e-8	9.04942e-7	2.9236	33	267	0.22%	21	54
	Motif NWATAAGTATWT matches NKX3-1: NK3 transcription factor related, locus 1 (Drosophila)	32	1.03760e-7	1.99413e-6	2.0762	61	695	0.41%	49	177
	Motif AGATAAGATAN matches EVI1: ecotropic viral integration site 1	53	1.01001e-6	1.17199e-5	2.0485	53	612	0.35%	35	147
	Motif TGCNHNCWYCCYCATTAKTNNDCNMNHYCN matches HOXA5: homeobox A5	90	1.05074e-5	7.18002e-5	2.8593	22	182	0.15%	15	31
	Motif TGACAGKTTTAYGA matches MEIS1: Meis1, myeloid ecotropic viral integration site 1 homolog (mouse)<br>HOXA9: homeobox A9	94	1.39560e-5	9.13080e-5	2.1014	39	439	0.26%	25	92
	Motif CCYCNRRSTNGCGTGASA matches AHR: aryl hydrocarbon receptor	109	2.88204e-5	1.62610e-4	2.3175	29	296	0.19%	16	65
	Motif CCTGAGAATAATC matches ALX4: aristaless-like homeobox 4	187	4.87106e-4	1.60198e-3	3.2937	11	79	0.07%	6	13
	Motif CRGAARNNNNCGA (no known TF)	283	4.06047e-3	8.82400e-3	2.1768	15	163	0.10%	15	35
	Motif NNNTNNNGNGTGANN matches PAX8: paired box gene 8	285	4.08169e-3	8.80787e-3	2.1144	16	179	0.11%	8	26
	Motif GCGSCMNTTT (no known TF)	324	7.74008e-3	1.46918e-2	2.0275	15	175	0.10%	10	49
MSigDB miRNA Motifs	Targets of MicroRNA TTGCACT,MIR-130A,MIR-301,MIR-130B	1	9.08124e-13	2.00695e-10	2.0072	121	1426	0.81%	82	328
	Targets of MicroRNA TAGCTTT,MIR-9	7	9.57895e-9	3.02421e-7	2.0669	72	824	0.48%	53	196
	Targets of MicroRNA TTGGAGA,MIR-515-5P,MIR-519E	9	1.71559e-8	4.21274e-7	2.3748	51	508	0.34%	36	121
	Targets of MicroRNA GACAGGG,MIR-339	11	2.71086e-8	5.44636e-7	2.8291	36	301	0.24%	21	53
	Targets of MicroRNA CTCTGGA,MIR-520A,MIR-525	13	6.92849e-8	1.17784e-6	2.1003	61	687	0.41%	40	131
	Targets of MicroRNA CAGCCTC,MIR-485-5P	20	9.15782e-7	1.01194e-5	2.0552	53	610	0.35%	39	116
	Targets of MicroRNA AACATTC,MIR-409-3P	21	1.24821e-6	1.31360e-5	2.2004	44	473	0.29%	30	117
	Targets of MicroRNA GTACAGG,MIR-486	35	5.57217e-6	3.51843e-5	2.9737	22	175	0.15%	14	48
	Targets of MicroRNA CCATCCA,MIR-432	40	8.90288e-6	4.91884e-5	2.6171	26	235	0.17%	15	51
	Targets of MicroRNA AAGGGAT,MIR-188	41	9.18552e-6	4.95122e-5	2.3808	31	308	0.21%	24	65
	Targets of MicroRNA ATCATGA,MIR-433	48	3.11490e-5	1.43415e-4	2.1744	33	359	0.22%	23	92
	Targets of MicroRNA TCTGATA,MIR-361	49	3.49919e-5	1.57820e-4	2.3743	27	269	0.18%	19	75
	Targets of MicroRNA ATTACAT,MIR-380-3P	55	7.40156e-5	2.97408e-4	2.2729	27	281	0.18%	20	85
	Targets of MicroRNA GCAAGAC,MIR-431	71	4.10532e-4	1.27785e-3	2.5451	17	158	0.11%	12	36
	Targets of MicroRNA AGGAGTG,MIR-483	72	4.54822e-4	1.39605e-3	2.0970	25	282	0.17%	16	59
	Targets of MicroRNA CGGTGTG,MIR-220	80	1.45010e-3	4.00591e-3	3.6392	8	52	0.05%	5	6
	Targets of MicroRNA CACGTTT,MIR-302A	89	2.47288e-3	6.14053e-3	2.7104	11	96	0.07%	10	24
	Targets of MicroRNA TCTAGAG,MIR-517	102	6.19342e-3	1.34191e-2	2.0239	16	187	0.11%	10	37
	Targets of MicroRNA TACGGGT,MIR-99A,MIR-100,MIR-99B	111	9.85266e-3	1.96165e-2	2.1668	12	131	0.08%	9	20
	Targets of MicroRNA GTCAACC,MIR-380-5P	123	1.72653e-2	3.10213e-2	2.4261	8	78	0.05%	7	19
InterPro	Molybdopterin synthase/thiamin biosynthesis sulphur carrier, beta-grasp	8	1.54242e-5	1.53239e-2	18.9237	4	5	0.03%	2	2
	Homeodomain protein CUT	9	1.61096e-5	1.42266e-2	4.3670	12	65	0.08%	6	7
	K Homology	10	1.68831e-5	1.34187e-2	2.8549	21	174	0.14%	12	39
	Lambda repressor-like, DNA-binding	11	3.35775e-5	2.42613e-2	2.6551	22	196	0.15%	12	28
	Pyridoxal phosphate-dependent decarboxylase	13	5.90158e-5	3.60813e-2	6.6233	7	25	0.05%	4	8
	Ubiquitin-related modifier 1	14	7.55387e-5	4.28844e-2	23.6546	3	3	0.02%	1	1
	LCCL	15	8.32193e-5	4.40951e-2	9.8561	5	12	0.03%	3	6
TreeFam	Zinc finger and BTB domain-containing protein 24	1	2.63778e-6	2.10732e-2	16.8961	5	7	0.03%	1	1
	CUX2/ONECUT2/ONECUT1/CUX1/ONECUT3	2	4.05736e-6	1.62071e-2	5.4208	11	48	0.07%	5	5
	Mucin-17 precursor	3	1.09606e-5	2.91880e-2	7.0088	8	27	0.05%	1	1

	Zinc finger and BTB domain-containing protein	4	1.65725e-5	3.30995e-2	7.8849	7	21	0.05%	4	4
HGNC Gene Families	CUT	1	1.61096e-5	7.90981e-3	4.3670	12	65	0.08%	6	7
	MUC	2	1.69869e-5	4.17029e-3	3.8065	14	87	0.09%	3	16
	ZBTB	3	8.61125e-5	1.40938e-2	2.2151	28	299	0.19%	16	41

The test set contains 14,977 (4%) of all 354,275 regions.	The test set picked 2,666 genes, the background set picked 14,129 genes.
GO Molecular Function has 3,402 terms covering 14,878 (84%) of all 17,635 genes.	3,402 ontology terms were tested (100%) using an annotation count range of [1, Inf].
GO Biological Process has 8,762 terms covering 14,731 (84%) of all 17,635 genes.	8,762 ontology terms were tested (100%) using an annotation count range of [1, Inf].
GO Cellular Component has 1,121 terms covering 16,374 (93%) of all 17,635 genes.	1,121 ontology terms were tested (100%) using an annotation count range of [1, Inf].
Mouse Phenotype has 7,294 terms covering 6,429 (36%) of all 17,635 genes.	7,294 ontology terms were tested (100%) using an annotation count range of [1, Inf].
Disease Ontology has 2,234 terms covering 7,911 (45%) of all 17,635 genes.	2,234 ontology terms were tested (100%) using an annotation count range of [1, Inf].
MSigDB Cancer Neighborhood has 427 terms covering 4,385 (25%) of all 17,635 genes.	427 ontology terms were tested (100%) using an annotation count range of [1, Inf].
PANTHER Pathway has 140 terms covering 2,183 (12%) of all 17,635 genes.	140 ontology terms were tested (100%) using an annotation count range of [1, Inf].
Pathway Commons has 1,607 terms covering 6,072 (34%) of all 17,635 genes.	1,607 ontology terms were tested (100%) using an annotation count range of [1, Inf].
MSigDB Pathway has 880 terms covering 6,686 (38%) of all 17,635 genes.	880 ontology terms were tested (100%) using an annotation count range of [1, Inf].
MGI Expression: Detected has 8,348 terms covering 11,006 (62%) of all 17,635 genes.	8,348 ontology terms were tested (100%) using an annotation count range of [1, Inf].
MSigDB Perturbation has 2,374 terms covering 16,270 (92%) of all 17,635 genes.	2,374 ontology terms were tested (100%) using an annotation count range of [1, Inf].
MSigDB Predicted Promoter Motifs has 615 terms covering 8,958 (51%) of all 17,635 genes.	615 ontology terms were tested (100%) using an annotation count range of [1, Inf].
MSigDB miRNA Motifs has 221 terms covering 6,031 (34%) of all 17,635 genes.	221 ontology terms were tested (100%) using an annotation count range of [1, Inf].
InterPro has 7,948 terms covering 16,411 (93%) of all 17,635 genes.	7,948 ontology terms were tested (100%) using an annotation count range of [1, Inf].
TreeFam has 7,989 terms covering 16,523 (94%) of all 17,635 genes.	7,989 ontology terms were tested (100%) using an annotation count range of [1, Inf].
HGNC Gene Families has 491 terms covering 6,911 (39%) of all 17,635 genes.	491 ontology terms were tested (100%) using an annotation count range of [1, Inf].

GREAT version 2.0.2
Species assembly: hg18
Association rule: Basal+extension: 5000 bp upstream, 5000 bp downstream, 0 bp max extension, curated regulatory domains included
